# Probing the impact of nairovirus genomic diversity on viral ovarian tumor domain protease (vOTU) structure and deubiquitinase activity

**DOI:** 10.1371/journal.ppat.1007515

**Published:** 2019-01-10

**Authors:** John V. Dzimianski, Brianna S. Beldon, Courtney M. Daczkowski, Octavia Y. Goodwin, Florine E. M. Scholte, Éric Bergeron, Scott D. Pegan

**Affiliations:** 1 Department of Pharmaceutical and Biomedical Sciences, University of Georgia, Athens, Georgia, United States of America; 2 Viral Special Pathogens Branch, Division of High Consequence Pathogens and Pathology, Centers for Disease Control and Prevention, Atlanta, Georgia, United States of America; Karolinska Institute, SWEDEN

## Abstract

Post-translational modification of host and viral proteins by ubiquitin (Ub) and Ub-like proteins, such as interferon stimulated gene product 15 (ISG15), plays a key role in response to infection. Viruses have been increasingly identified that contain proteases possessing deubiquitinase (DUB) and/or deISGylase functions. This includes viruses in the *Nairoviridae* family that encode a viral homologue of the ovarian tumor protease (vOTU). vOTU activity was recently demonstrated to be critical for replication of the often-fatal Crimean-Congo hemorrhagic fever virus, with DUB activity suppressing the type I interferon responses and deISGylase activity broadly removing ISG15 conjugated proteins. There are currently about 40 known nairoviruses classified into fourteen species. Recent genomic characterization has revealed a high degree of diversity, with vOTUs showing less than 25% amino acids identities within the family. Previous investigations have been limited to only a few closely related nairoviruses, leaving it unclear what impact this diversity has on vOTU function. To probe the effects of vOTU diversity on enzyme activity and specificity, we assessed representative vOTUs spanning the *Nairoviridae* family towards Ub and ISG15 fluorogenic substrates. This revealed great variation in enzymatic activity and specific substrate preferences. A subset of the vOTUs were further assayed against eight biologically relevant di-Ub substrates, uncovering both common trends and distinct preferences of poly-Ub linkages by vOTUs. Four novel X-ray crystal structures were obtained that provide a biochemical rationale for vOTU substrate preferences and elucidate structural features that distinguish the vOTUs, including a motif in the *Hughes orthonairovirus* species that has not been previously observed in OTU domains. Additionally, structure-informed mutagenesis provided the first direct evidence of a second site involved in di-Ub binding for vOTUs. These results provide new insight into nairovirus evolution and pathogenesis, and further enhances the development of tools for therapeutic purposes.

## Author summary

Viruses utilize a variety of mechanisms to manipulate and suppress host responses to infection. One specific mechanism used by nairoviruses is the production of a deubiquitinating enzyme, termed the vOTU, that disrupts the innate immune response. This enzyme has been shown to play a key role in efficient replication of Crimean-Congo hemorrhagic fever virus (CCHFV), a severe human pathogen causing outbreaks with high case fatality rates. Recent genomic studies have revealed a high degree of sequence variation for the vOTU among nairoviruses, but knowledge relating to the functional impact of this diversity is lacking. Here we investigated the effects of this diversity on the structure and function of vOTUs from a wide range of nairoviruses. This revealed that vOTUs from different nairoviruses possess distinct preferences for certain host proteins. In addition, we found that different vOTUs possess distinguishing structural features, including a unique motif present in one that was previously undescribed. Utilizing this information, we were able to provide a rational basis for the observed differences in the vOTUs. This work provides a foundation to understand nairovirus evolution by providing insight into a mechanism that influences virus host adaptation and pathogenesis.

## Introduction

**Fig 1:**
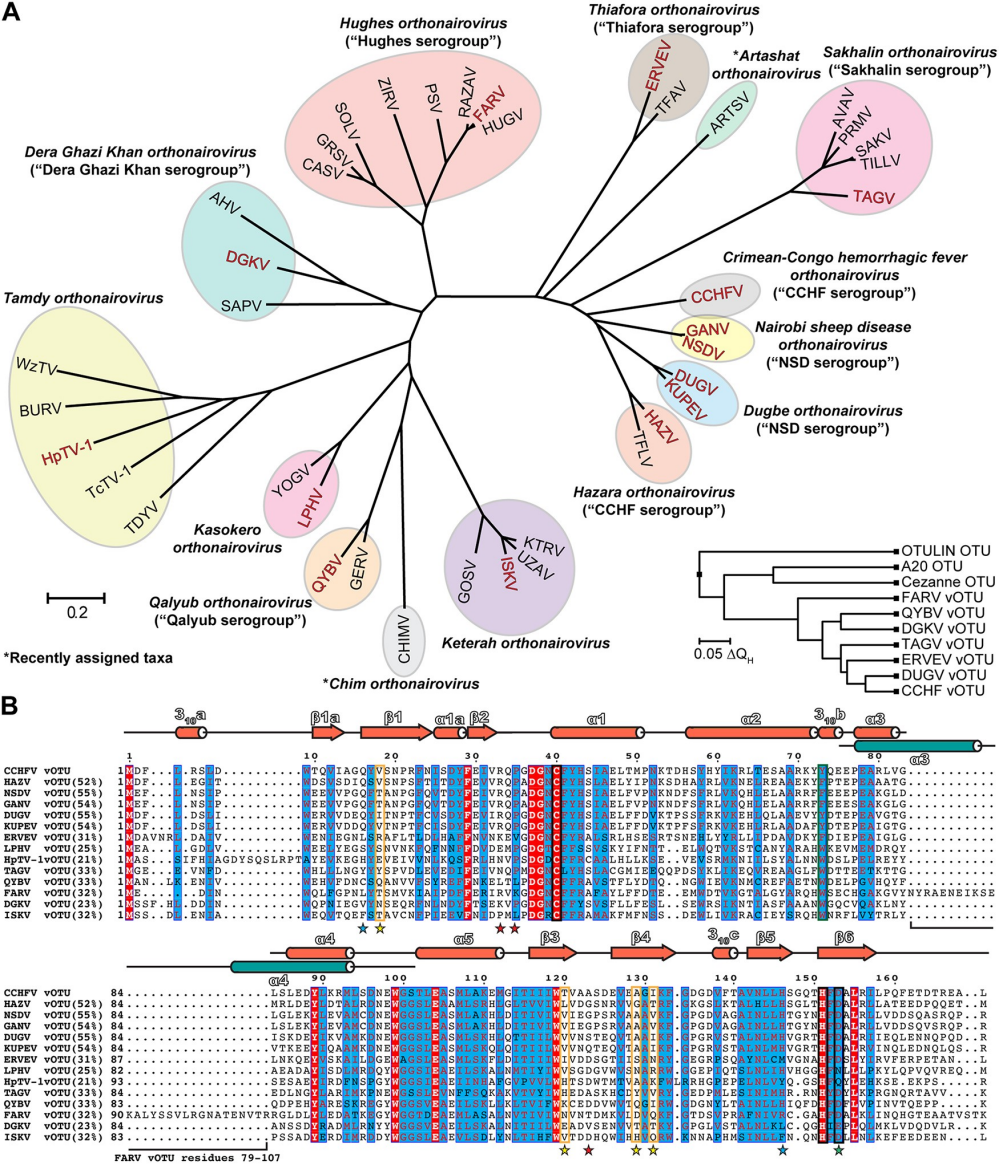
Sequence and structural diversity of nairovirus vOTUs. (A) Phylogenetic tree of CLUSTALW aligned nairovirus vOTUs. The tree was constructed utilizing the Jones-Thornton-Taylor model in the MEGA7 program [70]. Current species groupings are indicated by colored ovals, and the assigned species denoted. Previous serogroup classification, if applicable, is shown in parentheses. Virus vOTUs included in this study are denoted by red lettering. Inset is a structure-based phylogenetic tree of vOTUs, with the mammalian Cezanne, A20, and OTULIN OTUs included for comparison. The tree was constructed using PDB IDs 3PRP, 4HXD, 5JZE, 6DX1, 6DX2, 6DX3, 6DX5, 5LRV, 5LRX, and 3ZNZ in the MultiSeq module of VMD [71]. Sequence accession numbers are included in S1 Table. CCHFV, Crimean-Congo hemorrhagic fever virus; GANV, Ganjam virus; NSDV, Nairobi sheep disease virus; DUGV, Dugbe virus; KUPEV, Kupe virus; HAZV, Hazara virus; TFLV, Tofla virus; TAGV, Taggert virus; TILLV, Tillamook virus; SAKV, Sakhalin virus; PRMV, Paramushir virus; AVAV, Avalon virus; ARTSV, Artashat virus; TFAV, Thiafora virus; ERVEV, Erve virus; HUGV, Hughes virus; FARV, Farallon virus; RAZAV, Raza virus; PSV, Punta Salinas virus; ZIRV, Zirqa virus; SOLV, Soldado virus; GRSV, Great Saltee virus; CASV, Caspiy virus; AHV, Abu Hammad virus; DGKV, Dera Ghazi Khan virus; SAPV, Sapphire II virus; WzTV, Wēnzhōu tick virus; BURV, Burana virus; HpTV-1, Huángpí tick virus 1; TcTV-1, Tǎchéng tick virus 1; TDYV, Tamdy virus; YOGV, Yogue virus; LPHV, Leopards Hill virus; QYBV, Qalyub virus; GERV, Geran virus; CHIMV, Chim virus; GOSV, Gossas virus; ISKV, Issyk-kul virus; UZAV, Uzun-Agach virus; KTRV, Keterah virus. (B) Nairovirus vOTUs tested in this study aligned using the T-Coffee sequence alignment program [72]. Percentages show the sequence identity relative to CCHFV vOTU. Generic vOTU secondary structure based on Define Secondary Structure of Proteins (DSSP) algorithm calculations for the vOTUs is shown in reddish orange, with the α3 and α4 helices of FARV vOTU shown in teal. The catalytic triad is boxed in black and the selectivity pocket in orange. Mutation sites related to the selectivity pocket are shown by yellow stars, sites related to differences in how FARV vOTU engages mono-Ub by blue stars, and the DGKV vOTU catalytic triad mutant by a green star. Mutation sites for the second Ub binding site in FARV vOTU are denoted by red stars. The region deleted in the FARV vOTU^Δ79–107^ construct is indicated by a bracket.

Nairoviruses are negative sense single stranded RNA [(-) ssRNA] viruses within the order *Bunyavirales*. Initial classification of nairovirus species relied on antigenic cross-reactivity, leading to the clustering of viruses into seven serogroups; however, with the recent increase in the number of available viral sequences the classifications have shifted to a comparative genomics approach. This not only confirmed the diversity observed based on the serogroup classification, but also further accentuated how these viruses vary across the *Nairoviridae* family. The family *Nairoviridae* now consists of approximately 40 viruses that are currently classified into 14 species (Fig 1; [1–5]).

Most nairoviruses are tick-borne viruses infecting multiple vertebrate host species they parasitize in nature. Several have been implicated in human disease, the most notable being Crimean-Congo hemorrhagic fever virus (CCHFV), which has reported case fatality rates in humans that can exceed 30% [6]. Other nairoviruses associated with human disease include Dugbe virus (DUGV), Nairobi sheep disease virus (NSDV) and the Asian variant Ganjam virus (GANV), Erve virus (ERVEV), Issyk-kul virus (ISKV) and Kasokero virus (KASV). These viruses have been reported to cause a myriad of symptoms, some of which include fever, headache, and diarrhea [7–12]. Nairoviruses have also been observed to cause fatal animal disease. For example, NSDV has been reported to have a >90% mortality rate in sheep and goats making it a significant economic as well as human health concern [13]. A recently characterized nairovirus, Leopards Hill virus (LPHV), was isolated from bats and causes severe gastroenteric hemorrhaging and hepatic disease in mice [14]. Hazara virus (HAZV) was isolated from ticks collected from the Royle’s mountain vole and has been proposed as a model system to study CCHFV based on its ability to cause similar fatal disease in interferon (IFN)-receptor knockout mice [15, 16]. Beyond these viruses causing disease in mammalian hosts, other nairovirus have been associated with a broad taxonomic diversity of vertebrate hosts such as birds, fish, and reptiles. For example, viruses in the *Hughes orthonairovirus* species, such as Farallon virus (FARV), have been implicated in infecting birds [17].

Nairoviruses possess a tripartite genome consisting of small (S), medium (M), and large (L) segments that encode the viral nucleoprotein, glycoproteins, and RNA-dependent RNA polymerase, respectively. Interestingly, the nairoviral L segment also encodes a viral homologue of the ovarian tumor protease (OTU) at the N-terminus. This feature uniquely distinguishes the *Nairoviridae* family and genus *Tenuivirus* from other members of the order *Bunyavirales*. The viral OTU (vOTU) does not appear to play a direct role in genome replication and is dispensable in minigenome replication systems [18]. Instead, the vOTU’s primary function appears to be the reversal of post-translational modifications by ubiquitin (Ub) and the Ub-like protein interferon stimulated gene product 15 (ISG15). This vOTU-encoded deubiquitinase (DUB) and deISGylase activity has been implicated in evading the innate immune response [19–21]. Ub is an 8.5 kDa protein that is involved in a wide range of cellular processes, including key regulatory functions in innate immunity. Ub is conjugated to target proteins by means of a three step process involving activating (E1), conjugating (E2), and ligating (E3) enzymes, and can either occur as a single Ub moiety (mono-Ub) or in polymeric and branched forms (poly-Ub). These chains can be formed by linkage through either the N-terminus (linear) or one of seven lysine residues in Ub (K6, K11, K27, K29, K33, K48, and K63), with different forms often mediating different downstream effects. The most thoroughly studied forms, K48 and K63, play important roles in regulation of the innate immune responses. Specifically, K48-mediated proteasomal degradation has been associated with feedback control, while K63 polyubiquitination is required for pathway activation, including retinoic acid-inducible protein I (RIG-I), mitochondrial antiviral signaling protein (MAVS), Tumor necrosis factor (TNF) receptor associated factor 3 (TRAF3), TANK binding kinase 1 (TBK1), and IFN regulatory factor 3 (IRF3). This signaling cascade leads to the production of IFN-α/β, which ultimately results in the upregulation of numerous IFN-stimulated genes, including ISG15 [22, 23]. The role of ISG15 is complex and not well understood but is generally associated with mediating and regulating antiviral responses both as a co-translational modification and as free ISG15 in the cytosol and secreted form inducing the secretion of IFN-γ and IL-10 by binding cell surface receptor LFA-1 [24–29].

Initial studies on nairoviruses, including CCHFV, DUGV, and NSDV, established the potential immune modulatory effects of vOTU activity based on overexpression of the respective isolated OTU domain in cell culture [19–21]. The ability to probe the specific role of the vOTU during the viral replication cycle remained elusive, however, until the recent development of a reverse genetics system for CCHFV. These studies revealed distinct roles for DUB versus deISGylating activity during the course of a CCHFV infection [30]. Specifically, that CCHFV vOTU DUB activity is not as promiscuous towards ubiquitinated host proteins as it first seemed based on the overexpression studies, but appears to be restricted to a targeted subset of cellular substrates associated with suppression of RIG-I-mediated early cellular responses to infection. In particular, wildtype CCHFV was able to reduce the induction of several immune components, including RIG-I, while CCHFV with a vOTU specifically lacking DUB activity resulted in enhanced cellular responses to infection and establishment of a cellular antiviral state that reduced viral titers. In contrast, deISGylating activity appears to play a role in later stages of CCHFV infection. A recent study demonstrated a similar impact of DUB activity in viral immune suppression during the replication cycle of severe acute respiratory syndrome coronavirus (SARS-CoV) [31]. Specifically, when the DUB activity of the SARS-CoV papain-like protease (PLpro) was selectively disrupted, the virus showed increased sensitivity to IFN and slower growth kinetics. Furthermore, domain exchanges of PLpro’s between different SARS-CoV variants supported this observation, establishing DUB activity to be a distinguishing virulence trait. These emerging insights into the impact of DUB activity in the CCHFV vOTU and SARS-CoV PLpro during viral replication emphasizes the importance of robust DUB activity among pathogenic viruses. The demonstrated vOTU-associated DUB/deISGylase activity of other nairoviruses such as DUGV, ERVEV, and NSDV/GANV, further highlights a potentially substantial role of the vOTU in viral replication and immune suppression for viruses in the *Nairoviridae* family [19–21, 32].

Remarkably, the nairoviral vOTU domain shows a great degree of sequence diversity, with sequence identities that can drop below 25% between species (Fig 1). A particularly striking case of this diversity is found in members of the *Hughes orthonairovirus* species, such as FARV, which possess 26–30 additional residues in the middle of the OTU domain (Fig 1B). These sequence differences between vOTUs suggest a plasticity in the OTU domain that could play a role in evolutionary adaptation. Currently, exploration into the phenotypical effects of this diversity has been restricted to only a few taxa that include CCHFV, DUGV, NSDV/GANV, and ERVEV [32, 33]. These studies revealed that vOTUs possess different enzymatic and structural characteristics. In particular, vOTUs display a wide degree of variation in the efficiency with which they engage Ub and ISG15 that is driven by specific sequence and structural features. These substantial differences in viruses within closely related taxa raises questions on the impact of vOTU diversity across the *Nairoviridae* family. Specifically, how vOTUs from viruses in each species vary in structure and activity, and the implications of this for the potential to suppress the innate immune response and affect viral pathogenesis and host tropism.

To better understand the impact of vOTU diversity, we sought to obtain a more complete perspective of the functional and structural features of vOTUs within the *Nairoviridae* family. *In vitro* assays revealed that vOTUs across diverse taxa possess Ub activity, but that activity towards ISG15 appears more restricted. Further characterization of vOTU activity uncovered distinct trends and preferences for specific poly-Ub linkages. To better understand the molecular mechanisms driving Ub activity and specificity, novel X-ray crystal structures were solved revealing features that distinguish the vOTUs from each other, including a pocket that correlates with Ub specificity. Additionally, a structure of the FARV vOTU provides details into the structural nature of the additional residues in *Hughes orthonairovirus* vOTUs. Structure-informed mutagenesis of FARV vOTU identified residues involved specifically in di-Ub binding, representing the first report of the role of a second site involved in di-Ub binding in nairovirus vOTUs. This novel enzymatic and structural data not only provides insight into the nature of vOTU diversity, but also lays a foundation for understanding the impact of the vOTU interaction with the innate immune response and its connection to viral pathogenesis.

## Results

### vOTU enzymatic diversity

**Fig 2:**
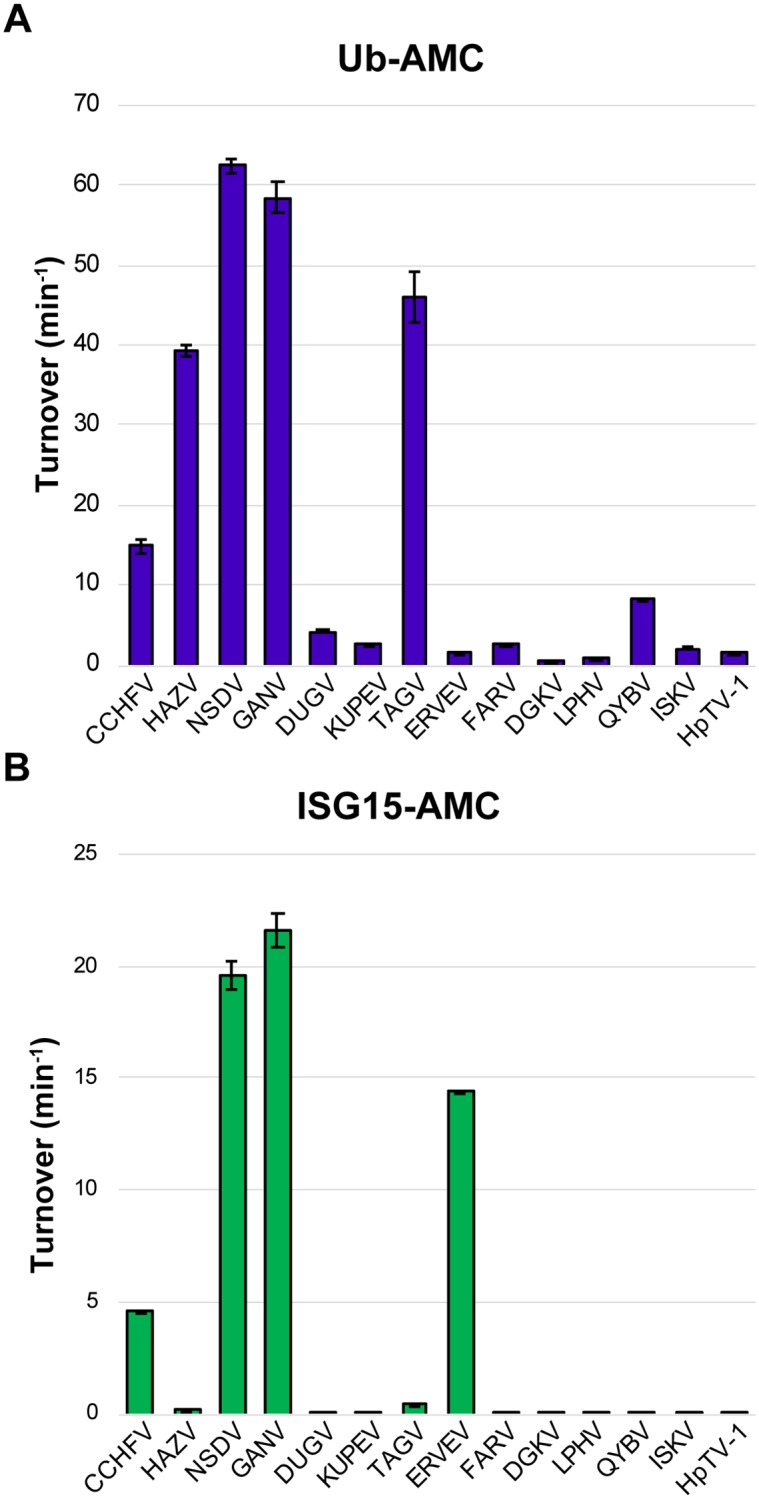
Diversity of vOTU specific activity. Activity of vOTUs towards towards Ub-AMC (A) and human ISG15-AMC (B). Values shown are the mean ± standard deviation of two independent experiments.

To gauge vOTU diversity across the *Nairoviridae* family, viruses representing the divergent species were selected and the OTU domain recombinantly expressed. Initially selected based on the traditional serogroups as well as emerging genetic characterization, these viruses include members of the most distantly related taxa and represent 12 of the currently recognized 14 species in the dynamic classification landscape of nairoviruses (Fig 1). Included were the vOTUs from CCHFV, NSDV and GANV, DUGV and Kupe virus (KUPEV), HAZV, Taggert virus (TAGV), ERVEV, FARV, Dera Ghazi Khan virus (DGKV), Huángpí Tick virus 1 (HpTV-1), LPHV, Qalyub virus (QYBV), and ISKV (Fig 1). To better understand the global diversity of nairoviral engagement with Ub and ISG15 substrates, these vOTUs were assessed for activity towards Ub and human ISG15 fluorogenic substrates. These specific activities were measured by the accumulation of the fluorescent molecule 7-amino-4-methylcoumarin (AMC) as a result of cleavage from the C-terminus of Ub or ISG15 (Fig 2).

Intriguingly, the vOTUs showed a diverse range of activity towards Ub. In general, vOTUs can be divided into groups possessing high (CCHFV, HAZV, NSDV/GANV, TAGV), moderate (DUGV, KUPEV, FARV, QYBV, ISKV), or low activity (ERVEV, DGKV, LPHV, HpTV-1) (Fig 2A). For some of these vOTUs, their deubiquitination activity mirrors that observed in DUB-deficient CCHFV mutants that impact cellular ubiquitination levels leading to an impaired ability to suppress the IFN response [30, 34]. To a large degree, viruses more closely related phylogenetically with CCHFV possess the most robust activity (Figs 1A and 2A). Beyond this, there is not an obvious phylogenetic trend to how well the vOTUs cleave Ub-AMC, with disparate taxa showing similar low to mid-range activity. Overall, engagement with Ub is observed to be a feature that can be present in diverse species in the *Nairoviridae* family, with some taxa demonstrating enhanced activity.

The patterns of activity for Ub are in stark contrast to those of ISG15-AMC, which shows a more dichotomous pattern as a substrate for the vOTUs (Fig 2B). Specifically, there appears to be an abrupt break phylogenetically between groups that contain vOTUs with deISGylating activity, compared to others for which activity is almost negligible. This break appears to exist at the node separating the *Thiafora*, *Artashat*, *Sakhalin*, *Crimean-Congo hemorrhagic fever*, *Nairobi Sheep Disease*, *Dugbe*, and *Hazara orthonairovirus* species from the remaining seven (Fig 1A). Interestingly, the presence of ISG15 activity does not encompass every vOTU in these species, suggesting individual factors may have driven the development or retention of ISG15 activity for viruses within this clade. Naturally, this also implies that DUB activity could be a more broadly utilized mechanism to evade cellular responses. This led us to further explore the dynamics of different nairovirus vOTU’s interactions with Ub.

### Di-Ub linkage preferences of vOTUs

**Fig 3:**
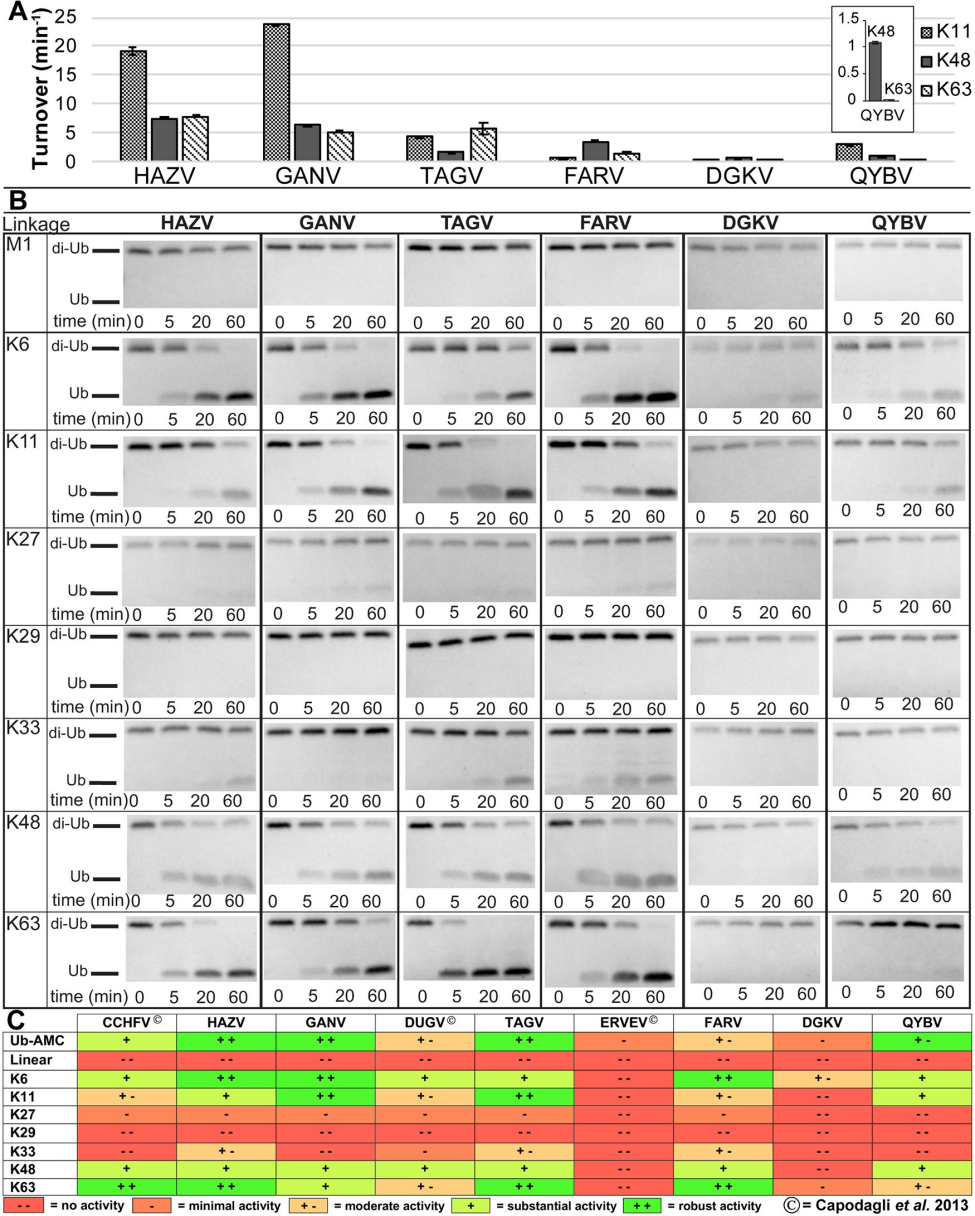
vOTU preferences for different di-Ub linkages. (A) Activity towards K48, K63, and K11 linked di-Ub FRET-TAMRA substrates. Values shown are the mean ± standard deviation of two independent experiments. (B) Gel cleavage assay of unlabeled di-Ub, visualized by Commassie Blue staining. (C) Summary of currently investigated vOTU di-Ub activity (present study and [32]).

While the Ub-AMC assay provides general information on the ability of vOTUs to engage monomeric Ub, cellular substrates are typically modified by poly-Ub chains through various linkage types [35]. Additionally, DUBs in general and vOTUs in particular have been observed to prefer some linkage types over others [32, 36–40]. To assess the patterns of Ub linkage preferences of diverse vOTUs, a subset of the vOTUs were analyzed against di-Ub FRET-TAMRA substrates (Fig 3A). HAZV vOTU and GANV vOTU were selected because they have been considered to be a potential model system for CCHFV and have significant health and economic impact, respectively. TAGV vOTU represents a more distantly related vOTU that also has substantial DUB activity, while the vOTUs encoded by QYBV, FARV, and DGKV display diminished activity towards mono-Ub. To reduce the influence of interactions with the FRET pairs that may disrupt interaction, multiple FRET pair configurations were assessed when available and the one displaying the highest activity selected (two positions each for K48 and K63; S1 Fig).

Comparison of vOTU activities towards different di-Ub FRET substrates reveals that each species’ vOTU has distinct preferences for specific di-Ub linkages. While HAZV vOTU and GANV vOTU both possess notable activity for K48 and K63 di-Ub, there appears to be more substantial activity towards K11. TAGV vOTU, on the other hand, prefers K63 and K11 to a greater extent, while not possessing as much activity towards K48. For FARV vOTU, the opposite is observed, with K48 being preferred. DGKV vOTU, consistent with its low Ub-AMC activity, possesses very low activity for the di-Ub FRET substrates, regardless of the linkage. Similar to the pattern observed for HAZV and GANV, QYBV vOTU shows the most activity towards K11, though at lower overall levels. Additionally, QYBV vOTU shows a more pronounced difference in the relative preference for K48 versus K63 linkages, with substantially more activity towards K48.

Regrettably, the commercial availability of FRET-TAMRA di-Ub substrates is restricted to these tested linkages. Additionally, limitations are known to exist due to how the positions of the donor-quencher pairs affects binding of these substrates with the proteases. To gain a more complete and natural perspective of di-Ub linkage preferences, these vOTUs were also assessed by SDS-PAGE for the ability to cleave unlabeled di-Ub substrates of all eight linkage types (Fig 3B). As expected, the vOTUs did not show equal preferences for the different linkages. Intriguingly, some of the results appeared to differ from the FRET analysis. Specifically, for both HAZV vOTU and FARV vOTU the gel cleavage assay would suggest the K63 activity to be the highest with K48 and K11 roughly equal, suggesting that the positions of the donor-quencher pairs may have hindered binding to some of the substrates. As expected, the HAZV, GANV, and TAGV vOTU showed substantial cleavage of several di-Ub substrates that is consistent with their high Ub-AMC activity, while the DGKV and QYBV vOTUs showed low level of substrate cleavage over the same time course. Intriguingly, FARV vOTU showed substantial cleavage of some of the substrates, despite not possessing high Ub-AMC activity. This may be reflective of differences in the assays in measuring DUB activity. Alternatively, it may also suggest the existence of an additional site of interaction with the proximal Ub that enhances the efficiency of di-Ub cleavage.

Assimilation with previously reported data reveals interesting trends and points of divergence between the vOTUs ([32]; Fig 3C). Linear and K29-linked di-Ub does not show any sign of cleavage with any vOTU. vOTUs demonstrate varying levels of low/detectable activity on K27-linked and K33-linked di-Ub. In contrast, vOTUs show a consistent pattern of higher activity towards K6, K11, K48, and K63 di-Ub. While most of the vOTUs show some level of enhanced activity towards these linkages, the specific linkage most preferred can differ. The CCHFV, HAZV, TAGV, and FARV vOTUs all show a distinct preference for K63 over K48 linkages, while DUGV and QYBV vOTU show more activity towards K48. GANV vOTU shows approximately equal preference for these linkages. Interestingly, GANV vOTU also shows more activity towards both K6 and K11 di-Ub at approximately equal levels. The high degree of preference towards these substrates extends to the majority of the vOTUs, as even for DGKV vOTU, which shows minimal or no cleavage of most of the substrates, cleavage of K6-linked di-Ub can be identified within an hour (Fig 3B). The other vOTUs, with the exception of ERVEV, all show detectable levels of K6 cleavage, with most also cleaving K11. The CCHFV, HAZV, DUGV, and FARV vOTUs all showed a greater relative preference for K6 over K11, while TAGV vOTU was the opposite. QYBV vOTU, similar to GANV, showed approximately equivalent activity towards K6 and K11, though overall activity was lower. Overall, these patterns of activity suggest that vOTUs do not merely cut any Ub moiety, but that they are specific to a subset of linkages that may influence specific aspects of cellular biology.

### Nairovirus vOTUs possess shared, but distinct structural characteristics

To gain a better understanding of how sequence diversity translates into structural differences, X-ray crystal structures were sought of vOTUs from divergent species. The vOTUs from DGKV, QYBV, and TAGV readily crystallized and were solved to 1.62 Å, 1.65 Å, and 2.05 Å, respectively (Table 1). These vOTUs represent diverse nairovirus species, and possess extensive variation in Ub activity with the DGKV, QYBV, and TAGV vOTUs possessing low, medium, and high activity towards Ub-AMC, respectively (Fig 2). TAGV provides a glimpse into the *Sakhalin orthonairovirus* species, a taxon that is more closely related to the ERVEV and CCHF/NSD/Dugbe/Hazara cluster, while DGKV and QYBV are from the *Dera Ghazi Khan* and *Qalyub orthonairovirus* species, respectively, and are much more distantly related (Fig 1).

**Table 1:** Data collection and refinement statistics for vOTU crystal structures.

	Se-SAD QYBV vOTU (PDB entry 6DWX)	QYBV vOTU (PDB entry 6DX1)	DGKV vOTU (PDB entry 6DX2)	TAGV vOTU (PDB entry 6DX3)	FARV vOTU (PDB entry 6DX5)
**Data collection**					
Space group	P3_2_21	P3_2_21	P4_1_	P1	P4_1_2_1_2
Wavelength (Å)	0.9795	1	1	1	1
Cell dimensions					
*a*, *b*, *c* (Å)	110.5, 110.5, 106.2	110.5, 110.5, 106.2	68.4, 68.4, 69.8	47.2, 60.9, 66.5	70.28, 70.28, 176.26
α, β, γ (°)	90, 90, 120	90, 90, 120	90, 90, 90	64.9, 89.8, 84.3	90, 90, 90
Resolution (Å)	50.00–2.40 (2.44–2.40)^†^	50.00–1.65 (1.68–1.65)^†^	50.00–1.62 (1.65–1.62)^†^	50.00–2.05 (2.09–2.05)^†^	50.00–2.22 (2.26–2.22)^†^
*R*_merge_	0.038 (0.118)	0.106 (0.821)	0.064 (1.071)	0.102 (0.450)	0.116 (0.874)
*CC*_*1/2*_	0.999 (0.996)	0.998 (0.719)	0.994 (0.568)	0.990 (0.847)	0.992 (0.890)
*I* / σ*I*	80.6 (29.9)	21.6 (1.8)	24.8 (1.1)	17.3 (2.3)	18.9 (2.9)
Completeness (%)	100.0 (100.0)	100.0 (100.0)	99.0 (99.5)	97.4 (97.7)	99.6 (100.0)
Redundancy	14.9 (15.0)	9.3 (6.2)	4.9 (4.3)	3.6 (3.4)	6.9 (6.8)
**Phasing statistics**					
No. of Se sites found	6				
Phasing Fig of merit	0.546				
**Refinement**					
Resolution (Å)	36.20–2.40 (2.48–2.40)	36.16–1.65 (1.71–1.65)	28.02–1.62 (1.67–1.62)	36.23–2.05 (2.13–2.05)	35.14–2.22 (2.30–2.22)
No. reflections	29,862	89,728	40,773	40,235	22,454
*R*_work_ (%)/ *R*_free_ (%)	0.174/0.195	0.171/0.186	0.180/0.212	0.227/0.250	0.163/0.203
No. atoms					
Protein	3843	3841	2492	5379	2730
Ligand/ion^‡^	0	0	0	2	24
Water	123	498	183	394	169
*B*-factors					
Protein	34.88	25.30	45.62	35.16	43.92
Ligand/ion^‡^	- -	- -	- -	55.16	103.21
Water	37.35	37.28	53.31	39.09	42.17
R.m.s. deviations					
Bond lengths (Å)	0.008	0.006	0.014	0.005	0.015
Bond angles (°)	0.83	0.76	1.26	0.71	1.24

^†^Values in parentheses denote the highest resolution shell

^‡^Includes Mg and DTT

**Fig 4:**
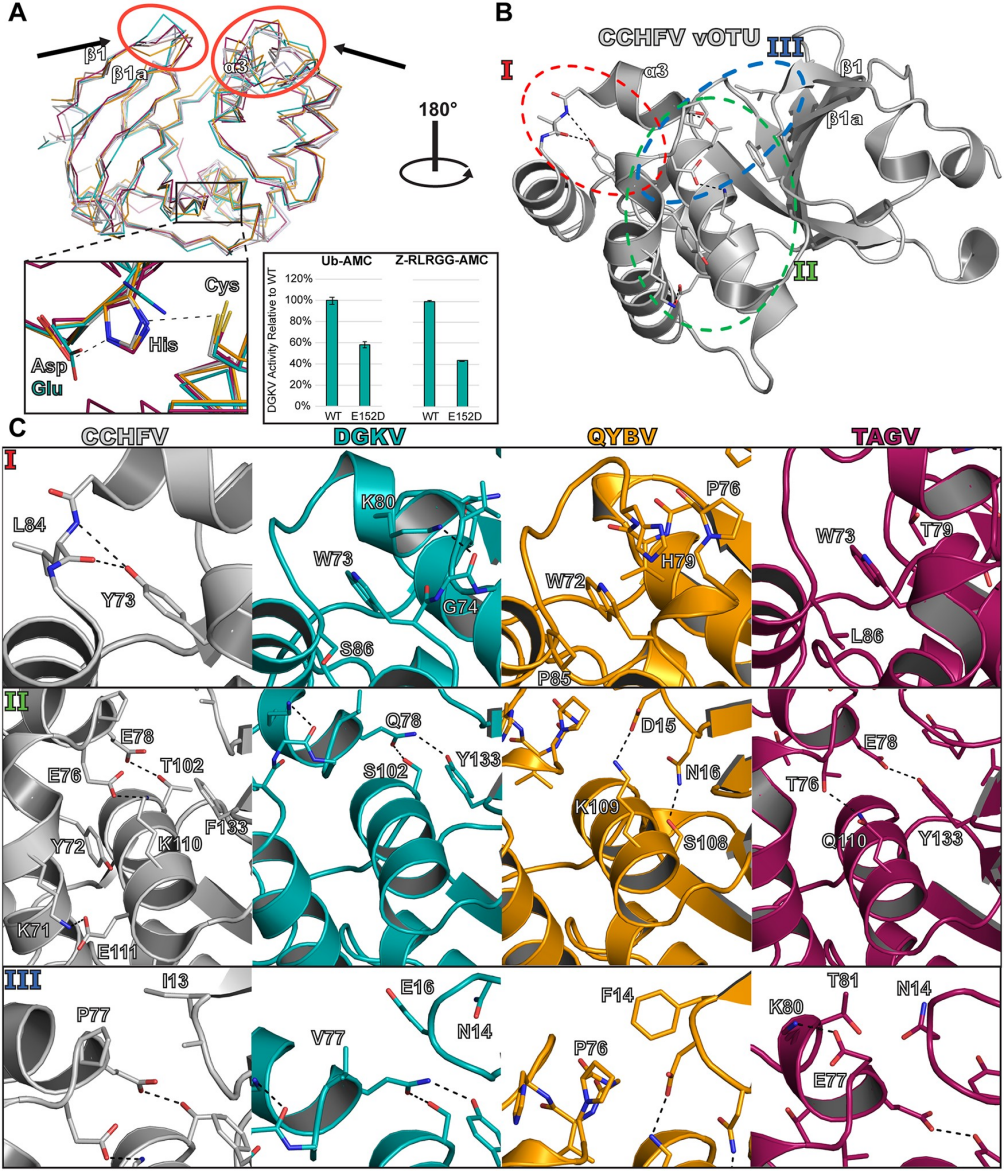
Structural comparison of new vOTU structures. (A) Secondary structure overlay of the vOTUs from CCHFV, DGKV, QYBV, and TAGV. The red circles highlight areas of the binding interface that show large structural divergence. The region of the active site is denoted by a black box, with a closeup shown. The relative activities of the DGKV vOTU WT and E152D mutant for Ub-AMC and the peptide Z-RLRGG-AMC are shown, with error bars representing the standard deviation of two independent experiments. (B-C) Specific molecular interactions accounting for structural differences can be identified in distinct regions of the vOTUs. CCHFV vOTU is colored gray, DGKV vOTU in teal, QYBV vOTU in orange, and TAGV vOTU in magenta. Black dashed lines denote atom pairs that are within hydrogen bonding distance.

These structures reveal global similarities among the vOTUs (Fig 4 and S2 Fig). Each vOTU possesses a seven-stranded beta sheet as the core feature, with five major alpha helices framing the rest of the structure. The catalytic triads perfectly superimpose over each with the exception of DGKV vOTU (Fig 4A). In DGKV vOTU, aspartate is replaced by a glutamate that alters the spatial dynamics of the catalytic triad, possibly contributing to a less rigid structure that allows the histidine to adopt the alternate conformation. While atypical for the vOTUs, it does not appear to be the cause of DGKV’s low DUB activity, as mutating the glutamate to an aspartate only further diminished activity. Looking beyond the catalytic triad, a structural overlay of the vOTUs highlights a point of difference in the overall structure that distinguishes the proteases from each other. Specifically, there appears to be substantial variability in the region encompassing the α3 (“selectivity”) helix that has been associated with substrate preference, and the loop between the β1 and β1a strands (Fig 4A; [32, 33]). Comparing the root mean square deviation (R.m.s.d.) for the positions of the main chain atoms of these different structures further emphasizes how they deviate from structure to structure (S2 Fig).

Closer examination of the structures reveals distinct molecular interactions that account for these observed structural differences within the vOTUs. Specifically, particular amino acid differences can be identified that form interactions that would promote the observed conformation of each protease, suggesting these differences to not merely be a consequence of dynamics or crystal packing. Intriguingly, these residues are not limited to just the selectivity helix and β1-β1a loop but extend to other secondary structural elements in local proximity, forming an ensemble of interactions that drive the noticeable variability of the α3 region (Fig 4B and 4C). The first area of prominent influence centers around position 73 of CCHFV vOTU (Fig 4C, Panel I). This position is strongly conserved in possessing an aromatic residue, consisting of a phenylalanine or a tyrosine in CCHFV and more closely related viruses, while consisting of a tryptophan in the rest of the vOTUs studied (Fig 1B). While subtle, this change results in distinctly different local interactions that influence the positioning of the selectivity helix. In the CCHFV vOTU, Tyr73 forms hydrogen bonds with the backbone of Leu84 within the α3-α4 loop. In contrast, the tryptophan residues in the other vOTUs fill into a hydrophobic cleft that involves residues within the selectivity helix. In DGKV vOTU, Trp73 packs with the methylene group of Ser86 on one side and the aliphatic portion of Lys80’s side chain on the other. Lys80 itself is stabilized in this permissive conformation by hydrogen bond pairing with the carbonyl of Gly74. In QYBV vOTU, Trp72 packs with Pro85. Additionally, it is in proximity to His79, suggesting potential stacking of the rings. Such an interaction could have an indirect effect on the positioning of Pro76 at the surface of the vOTU-substrate interface. In TAGV vOTU, Trp73 fits between Leu86 and Thr79.

The second area centers around helix α5 and shows a great degree of variation between the vOTUs (Fig 4C, Panel II). It can encompass interactions that can extend to the α2 and α3 helices as well as the β1a sheet with the potential to influence the local structural architecture. CCHFV vOTU possesses a number of interactions within this region, including unique lysine pairings consisting of Lys71, Glu111, Lys110, and Glu76 that accommodate hydrophobic packing with Tyr72 and Phe133. Along with hydrogen bond pairing of Glu78 with Thr102, these work in conjunction in orienting the position of the selectivity helix, a region that has been implicated in substrate preference [32, 34]. The other vOTUs, in contrast, possess fewer interactions but still could influence the structure. In DGKV vOTU, Gln78 within the selectivity helix is central to the interaction, pairing with both Ser102 and Tyr133 by hydrogen bonding. Similarly, for TAGV vOTU Thr76 and Glu78 form electrostatic interactions with Gln110 and Tyr133, respectively. In contrast, for QYBV vOTU there do not appear to be any direct interactions with the selectivity helix. Instead, Lys109 and Ser108 form electrostatic interactions with Asp15 and Asn16, respectively, suggesting a role in manipulating the positioning of the β1-β1a loop.

The third region consists of the selectivity helix and β1-β1a loop themselves (Fig 4C, Panel III). Specifically, direct interactions, or the lack thereof, work in conjunction with the other interactions to complete the structural features. This is most notable in QYBV vOTU, in which Phe14 appears to pack with Pro76. In CCHFV vOTU, the corresponding residue is Ile13, which does not appear to be able to bridge the distance and form an interaction. In TAGV vOTU, Asn14 and Thr81 are in general proximity, but appear to be too distant to form a strong interaction with each other. Similarly, DGKV vOTU appears to lack any direct interactions. Overall, these interactions contribute to structural features that influence spatial and chemical presentation of the vOTU interface, potentially affecting how these vOTUs engage substrates.

### General deubiquitinating activity can be correlated to a “selectivity pocket”

**Fig 5:**
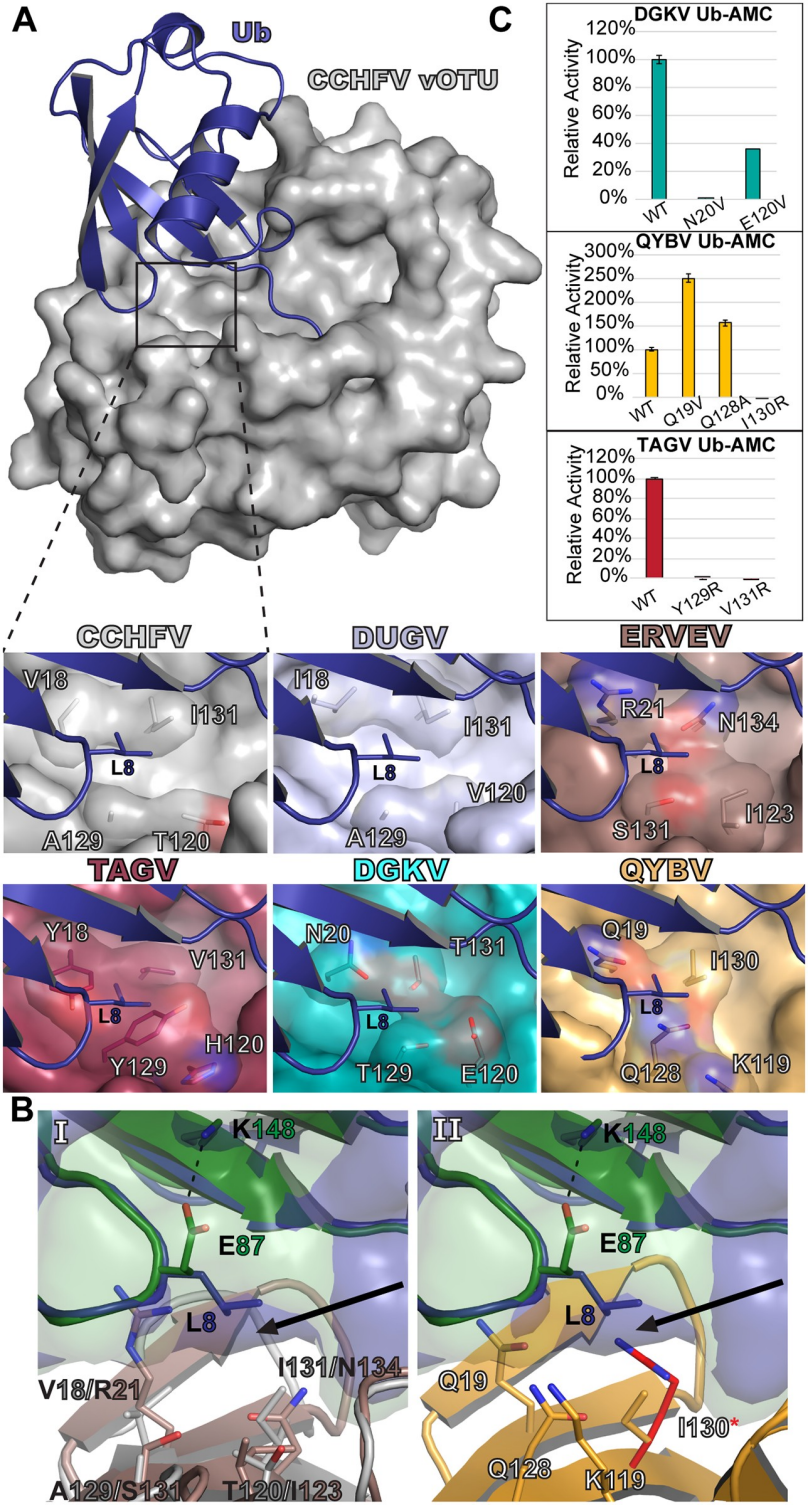
Selectivity pocket of nairovirus vOTUs. (A) The CCHFV vOTU Ub bound structure (PDB ID 3PRP) is shown with the location of the selectivity pocket indicated with a box. The selectivity pockets of the other vOTUs are shown, with the Ub (purple) modeled in from the CCHFV structure based on a secondary alignment of the vOTUs. DUGV vOTU (PDB ID 4HXD) is colored silver and ERVEV vOTU (PDB ID 5JZE) is colored brown. The other vOTUs are colored as in Fig 3. All models were generated by aligning the vOTUs with the CCHFV vOTU Ub bound structure in Coot. (B) The extra space (black arrow) existing in the ERVEV-mouse ISG15 structure (PDB ID 5JZE), with the CCHFV vOTU bound to Ub included for comparison (Panel I). QYBV vOTU with Ub and mouse ISG15 (green) modeled in based on vOTU secondary structure alignments, with an arginine also modeled into the space opened up by the mouse ISG15 conformation (Panel II). (C) Activities for Ub-AMC for mutant DGKV, QYBV, and TAGV vOTUs relative to WT. Error bars represent the standard deviation of two independent experiments.

Binding with Ub often centralizes around the specific hydrophobic residues Leu8, Ile44, and Val70 [41]. Looking at X-ray crystal structures of the CCHFV and DUGV vOTUs bound to Ub reveals that Leu8 in particular has to be spatially accommodated in a pocket deep within the interaction interface (Fig 5A). To confirm that this interaction with Leu8 is likewise involved in Ub binding with these other vOTUs, isothermal titration calorimetry (ITC) was performed using the TAGV vOTU to determine the relative binding efficiency of alanine and asparagine Ub mutants (Ub-L8A and Ub-L8N) compared to WT Ub (Table 2, S3A Fig). This revealed a stark difference in the affinity. While WT Ub bound strongly with a dissociation constant (K_D_) of 11.5 ± 2.5 μM, Ub-L8A showed no detectable binding under similar conditions. The Ub-L8N mutant faired only slightly better than the Ub-L8A mutant with a 20 times weaker dissociation constant, K_D_ of 295.3 ± 39.7 μM, compared to WT. These results further underscore the importance of vOTUs being able to accommodate Ub Leu8 in order to have robust deubiquitinating activity. Examining the analogous residues in the other vOTUs reveals a diverse composition for this pocket that could influence how well Leu8 can be accommodated. In TAGV vOTU, this pocket is largely hydrophobic possessing two tyrosines as well as a valine. In contrast, the DGKV and QYBV vOTUs possess more polar residues, including asparagine, glutamate, and threonine for DGKV and glutamine and lysine in QYBV. When considering the activity towards Ub based on the AMC assay, a general trend emerges that correlates the degree of an enzyme’s ability to engage mono-Ub with the hydrophobicity of this pocket.

**Table 2:** Isothermal Titration Calorimetry of TAGV-Ub binding.

Protein	N^a^	K_D_	ΔH^b^	ΔG^c^	-TΔS^d^
	(sites)	(μM)	(kJ/mol)	(kJ/mol)	(kJ/mol)
Ub^e^	1.34 ± 0.09	11.5 ± 2.5	-19.6 ± 1.5	-28.3 ± 0.6	-8.6 ± 2.0
Ub-L8A^e^		>1 mM			
Ub-L8N^e^	0.99 ± 0.14	295.3 ± 39.7	-11.0 ± 2.5	-20.2 ± 0.3	-9.2 ± 2.9

^*a*^ Binding stoichiometry.

^*b*^ Binding enthalpy.

^*c*^ Gibb’s free energy.

^*d*^ Entropy factor.

^*e*^ Average from n = 3 with error calculated using the standard deviation

Looking more closely at this interface suggests an additional nuance to the ability to accommodate particular substrates. Specifically, spatio-chemical characteristics could largely influence what defines a good or acceptable pocket composition for binding a given substrate. In the vOTUs that most effectively engage with Ub, such as CCHFV, HAZV, NSDV/GANV, and TAGV, the residue that most directly interfaces with Ub’s Leu8 is an isoleucine, valine, or threonine that corresponds to position 131 in CCHFV vOTU (Figs 1B, 2 and 5A). This correlation is consistent across the *Nairoviridae* family. Despite being phylogenetically distant from CCHFV and the other robust vOTU DUBs, QYBV demonstrates substantial Ub activity and possesses Ile130 that could pack with Ub’s Leu8.

In other vOTUs that have poor Ub activity this residue is typically polar, such as ERVEV’s Asn134 (Fig 5B, Panel I). This creates an environment that would discourage binding with Ub. Mutation of this residue in ERVEV to the corresponding hydrophobic residues in CCHFV has been observed to generate robust Ub activity [33]. The FARV and ISKV vOTUs appear to have similar characteristics, with both encoding a glutamine at this position. Intriguingly, other vOTUs may go even further in discouraging Ub binding. These include LPHV and HTV-1, which possess an arginine and lysine, respectively, at their equivalent positions to Asn134. Modeling in an arginine at this position, such as what LPHV vOTU possesses, reveals that this type of change would be prohibitive for Ub binding (Fig 5B, Panel II).

To test the central role of this pocket for Ub activity, a series of mutants were made in the DGKV, QYBV, and TAGV vOTUs and tested against Ub-AMC (Fig 5C). As expected, the disruptive mutants I130R in QYBV and Y129R and V131R in TAGV completely knocked out the ability to process Ub-AMC, in keeping with a previous report demonstrating that the presence of arginine hindered ERVEV vOTU Ub activity [33]. Further, increasing the hydrophobicity of the pocket in QYBV vOTU was able to enhance Ub-AMC cleavage, boosting activity by 150% and 50% for the Q19V and Q128A mutants, respectively. Interestingly, changing the pocket in DGKV vOTU failed to improve activity. This suggests that some vOTUs lacking any outright DUB activity may have evolved to a degree that prevents the generation of this activity through simple changes to better accommodate Leu8 in Ub. This leaves the presence of a Ub Leu8 accommodating pocket in vOTUs as a major marker for deubiquitinating activity and, if present, the ability to dictate variable levels of activity based on the hydrophobicity.

### *Hughes orthonairovirus* vOTUs possess a unique coiled-coil structural feature

**Fig 6:**
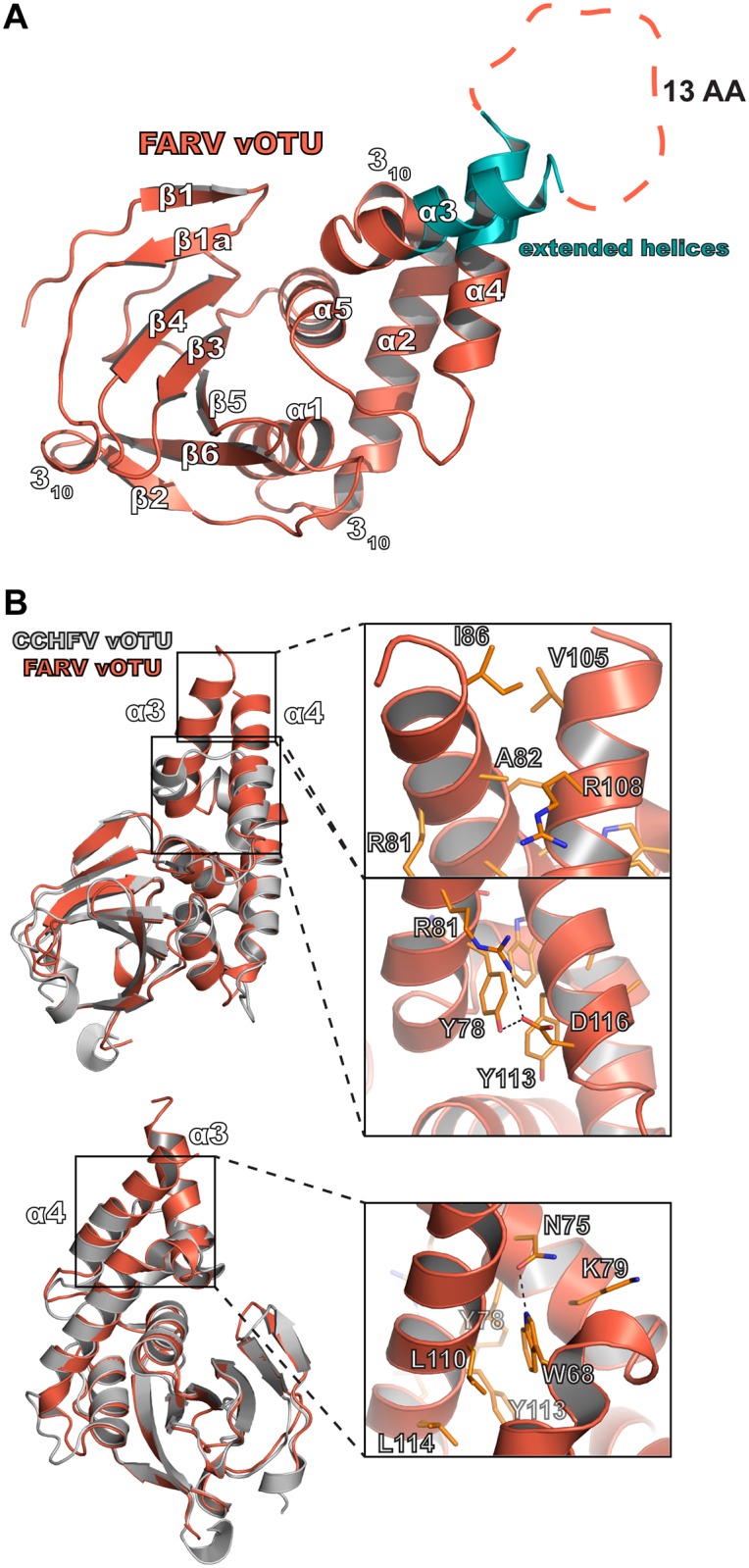
Structure of the FARV vOTU. (A) Overall structure of the FARV vOTU with the secondary structure denoted based on DSSP. The extended regions of the α3 and α4 helices are colored in teal. Intervening amino acids lacking electron density are represented by an orange dashed line. (B) Molecular features of the extended α3-α4 helices of FARV vOTU, with CCHFV vOTU included for comparison. Atom pairs within hydrogen bonding distance are denoted by black dashes.

While the vOTUs show a range of sequence diversity, most of them possess domains of approximately the same size with one notable exception. Viruses in the *Hughes orthonairovirus* species possess an additional 26–30 amino acids in the middle of the vOTU. When aligned with the other vOTUs, this extra sequence corresponds to the region of the α3/α4 helices. To assess how this additional sequence influences the structure of vOTUs in this subset of nairoviruses, a crystal structure of FARV vOTU was solved to 2.22 Å (Table 1). Inspection of the structure immediately revealed the impact of this additional sequence on the protease’s structure (Fig 6A). While possessing the familiar core domain and secondary structure features, the protease possesses extended α3/α4 helices that are connected by several intervening residues. Thirteen residues could not to be built due to a lack of well-defined electron density in the crystal structure, and those that could be modeled possess high B factors, suggesting this region to have a high degree of flexibility. This contrasts with the vOTUs from CCHFV and other nairoviruses that possess relatively small α3/α4 helices connected by a short loop (Fig 6B). Additionally, the α3/α4 helices of FARV vOTU appear to interact with each other in a manner resembling a coiled-coil motif. This is facilitated by hydrophobic packing between Ile86, Val105, Ala82, and the aliphatic portion of the Arg108 sidechain. This relationship between the helices is further promoted by electrostatic interactions, including a salt bridge between Arg81 and Asp116, as well as a hydrogen bond between Tyr78 and Asp116. Beyond this interaction, Tyr78 is also positioned to hydrophobically interact with Tyr113, which together create an environment in which Trp68 can insert. Trp68 further promotes the interaction between these helices through a hydrogen bond with Asn75, as well as through additional hydrophobic packing with Lys79 and Leu110.

**Fig 7:**
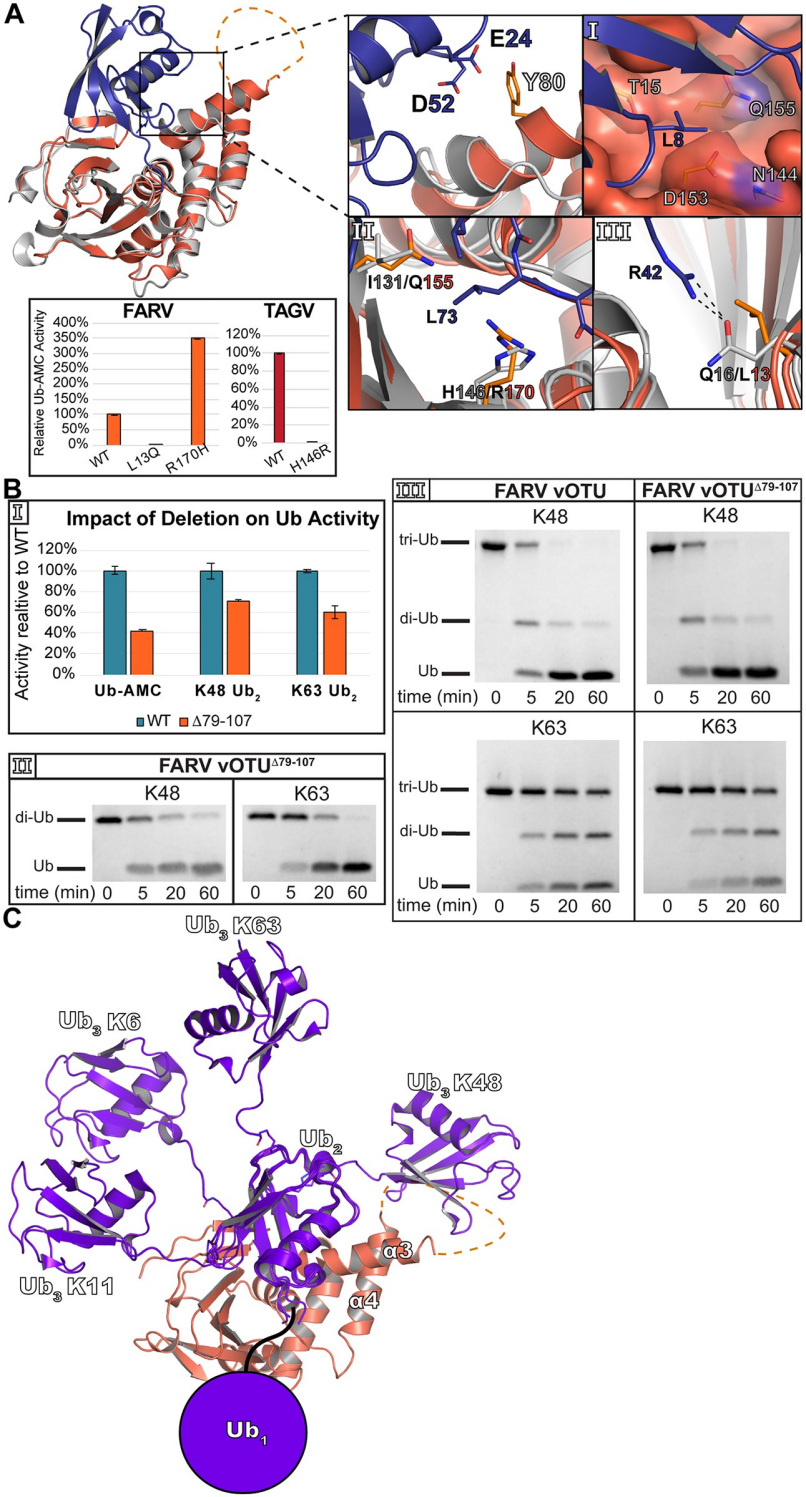
Model of molecular contributors to FARV mono- and poly-Ub activity. (A) FARV vOTU (reddish orange) in a Coot-calculated secondary structure overlay with CCHFV vOTU (gray) bound to Ub (PDB ID 3PRP). The selectivity pocket of FARV vOTU is shown in Panel I, with other elements potentially diminishing FARV vOTU Ub activity in Panels II and III. Black dashes show hydrogen bond interactions. Inset shows the Ub-AMC activity relative to WT of FARV and TAGV vOTU mutants. Error bars represent the standard deviation of two independent experiments. (B) Enzymatic activity of FARV vOTU^Δ79–107^ compared to WT for Ub-AMC and K48/K63 FRET-TAMRA (Panel I), gel cleavage assays of K48/K63 di-Ub with FARV vOTU^Δ79–107^ (Panel II), and gel cleavage assays of K48/K63 tri-Ub with WT FARV vOTU and FARV vOTU^Δ79–107^ (Panel III). (C) Model of tri-Ub binding with FARV vOTU. The proximal Ub of K6 linked (PDB ID 5OHP), K11 linked (PDB ID 5LRV), K48 linked (PDB ID 5E6J), and K63 linked (PDB ID 2JF5) di-Ub was anchored to bound mono-Ub based on a secondary structure alignment in Coot. The filled circle indicates the common space that would likely be occupied by the Ub interacting with the second site of interaction of FARV vOTU.

The presence of the extra sequence/structural motif in the *Hughes orthonairovirus* species raises the question of whether it could be involved in substrate interaction. A model of how FARV vOTU could interact with Ub further accentuates this possibility, suggesting the α3/α4 helices to be in close enough proximity to participate in binding (Fig 7). Such an interaction could potentially offset other factors in FARV vOTU are not ideal for binding. Looking at the selectivity pocket of FARV vOTU reveals it to possess more of a hydrophilic character and contains a relatively bulky Gln155 residue in the equivalent site to position 131 in CCHFV (Fig 7A, Panel I). Additionally, FARV vOTU possesses a potential steric hindrance to efficient binding with the presence of Arg170 (Fig 7A, Panel II). This residue may be less accommodating for the Leu73 in Ub than other vOTUs, such as CCHFV and TAGV which contain a histidine at this site. Further, FARV vOTU may lack a significant interaction that CCHFV vOTU possesses with Arg42 of Ub (Fig 7A, Panel III). In contrast to Gln16 in CCHFV vOTU that is able to form a hydrogen bond, FARV vOTU possesses a leucine that is unable form this interaction. To test the influence of these sites on DUB activity, mutations were made to Arg170 and Leu13 in FARV vOTU to histidine and glutamine, respectively. As anticipated, R170H was able to improve Ub-AMC activity, boosting it by ~250%. Making the reverse mutation in TAGV vOTU, H146R, essentially knocked out this activity suggesting this residue to have a key impact in diminishing FARV vOTU’s activity compared to other vOTUs. Interestingly, the L13Q mutation in FARV vOTU led to a large reduction in Ub-AMC cleavage. Looking more closely at this region shows that Leu13 is in the middle of a large hydrophobic region in FARV vOTU (S3B Fig). The swap to a large polar residue may impact the structural integrity of the β1-β1a region, further underscoring the nuances created by the variability of this region.

To probe the potential significance of the α3/α4 motif in offsetting these other effects in FARV vOTU, a construct was synthesized lacking residues 79–107 (“FARV vOTU^Δ79–107^”) and assessed for activity against Ub substrates (Fig 7B). Removing this region reduced activity towards Ub-AMC by almost 60%, suggesting that this motif could play a significant role in Ub binding (Fig 7B, Panel I). Interestingly, when tested against K48 and K63 FRET di-Ub substrates a more modest reduction in activity is observed, with only about a 30% and 40% reduction in activity, respectively. This is further borne out with unlabeled di-Ub, with there being no substantial difference between the WT and Δ79–107 vOTUs over the longer reaction time course (Figs 3B and 7B, Panel II).

Although the di-Ub cleavage assays are able to differentiate linkage preference, the structural architecture is still relatively simple. To gauge whether this motif can engage with more complex poly-Ub structures, the WT and Δ79–107 FARV vOTUs were tested with K48 and K63 linked tri-Ub (Fig 7B, Panel III). Interestingly, both constructs showed a clear preference for K48 over K63 tri-Ub. This is in contrast to the gel cleavage assay for di-Ub, which showed a slight preference for K63 di-Ub (Figs 3B and 7B, Panel II). Beyond this, both constructs showed similar patterns of activity for these substrates.

### Activity towards di-Ub is influenced by residues outside the central Ubiquitin Interacting Motif

**Fig 8:**
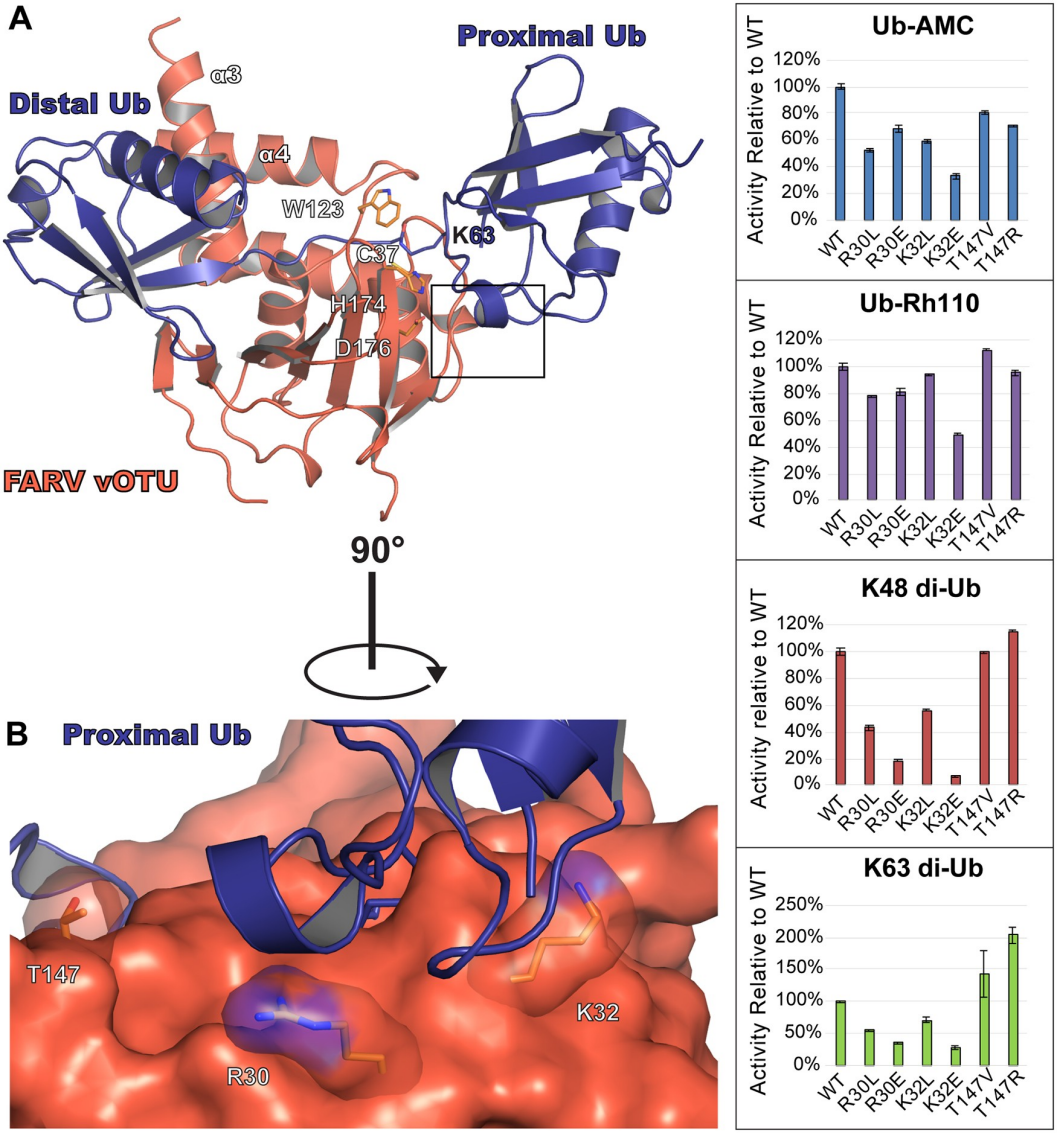
Second site of FARV vOTU interaction with di-Ub. (A) Model of FARV vOTU (reddish orange) bound to K63-linked di-Ub (purple; PDB ID 2JF5). FARV vOTU was overlaid with CCHFV vOTU bound to Ub (PDB ID 3PRP; not rendered) based on secondary structure alignment of the vOTUs. The distal Ub was anchored to the bound mono-Ub by aligning the secondary structure in Coot, followed by manual bond rotations within Lys63 of the proximal Ub in PyMol to model a plausible fit with minimal clashes based on the CCHFV vOTU active site and protease surface. The predicted region of FARV vOTU engagement with the proximal Ub is indicated by a black box. (B) Closeup view of the predicted region, with the residues selected for mutation shown as sticks. Activity of the mutants relative to WT is shown for Ub-AMC, Ub-Rh110, K48 di-Ub FRET-TAMRA, and K63 di-Ub FRET-TAMRA (right).

Despite possessing low to moderate activity towards Ub-AMC, FARV vOTU possesses substantial activity towards some di-Ub linkages (Figs 2 and 3). This suggests an additional site of interaction with the proximal Ub molecule that substantially increases the overall efficiency. To ascertain where this site may be located, a model of how FARV vOTU may bind di-Ub was generated (Fig 8). Examining the potential interface with the proximal Ub, two residues in FARV vOTU, Arg30 and Lys32, immediately stand out as potential contributors. These residues are just beyond the active site, and are part of a region that likely forms the closest contact with the proximal Ub. Beyond these two residues, Thr147 of FARV vOTU also stands out as being in an area with a higher R.m.s.d. between the vOTU structures, which in FARV vOTU positions it closer to the general area of the proximal Ub (Fig 1B and S2 Fig).

To assess whether these sites could play a role in the FARV vOTU’s interaction with di-Ub, mutations at these positions were designed in an attempt to alter activity towards K48 and K63 FRET di-Ub substrates. As a control, each mutant was also run with mono-Ub substrates. Due to the proximity of Arg30 and Lys32 to the space that would be occupied by the fluorogenic molecule, assays were performed with both Ub-AMC and Ub-Rhodamine110 (Rh110) to mitigate artifacts. Excitingly, these mutations substantially altered the rate of di-Ub cleavage, often towards both substrates (Fig 8). Individually mutating Arg30 and Lys32 to leucine reduces activity towards K48 di-Ub to 43–55% of wildtype and activity towards K63 to 56–70%. Although the mono-Ub activity appears to suffer as well in the case of R30L, the ~30% difference in the Ub-AMC versus Ub-Rh110 mono-Ub substrates suggests this to be an artifact of interactions with the AMC fluorophore. Otherwise, these mutants had little or no effects on mono-Ub activity. When a charge flip was introduced at position 30, activity was reduced to 18% for K48 and 35% for K63 while not substantially altering the activity for mono-Ub. A charge flip at position 32 had the most pronounced effect, driving it down to 7% for K48 and 38% for K63. However, there is also a substantive corresponding reduction in both the AMC and Rh110 mono-Ub substrates, indicating a potentially large disruptive interaction with the hydrophobic fluorophores or possible influence on the local fold. Interestingly, while mutating Thr147 to valine did not appreciably change the activity, introducing an arginine at this position increased the activity by 15% for K48 and doubled it for K63, suggesting the longer sidechain may be able to form a new interaction.

## Discussion

### Taking advantage of Ub conservation

Ub is among the most conserved and important cellular regulatory components, influencing almost every key aspect of cell biology. Ub itself is tightly regulated by an array of endogenous DUB enzymes that specifically curb and tailor its effects. The realization that viruses also possessed enzymes with DUB activity introduced a paradigm in which these normal regulatory mechanisms could be manipulated to suppress immune responses and enhance viral propagation [19, 42–45]. Further investigation into these mechanisms continues to uncover how these viral DUBs disrupt cellular responses to infection. In particular, the role of robust DUB activity in promoting viral replication and conferring virulence in CCHFV and SARS-CoV emphasizes the impact of the respective proteases and highlights the emerging importance of understanding their effects when considering potential pathogenicity and therapeutic strategies.

With the almost perfectly conserved sequence of Ub, it is not surprising that tick-borne nairoviruses from disparate taxa possess notable DUB activity. Such a mechanism could provide broad utility in infecting hosts beyond the primary arthropod reservoir by enabling a route of horizontal as well as vertical transmission that amplifies viral replication. The diversity in the observed activity, however, raises questions as to the specific effects relating to arthropod versus vertebrate hosts. In general, the vOTUs from viruses most closely related to CCHFV appear to have the most substantial DUB activity based on the Ub-AMC assay (Figs 1 and 2). These viruses are known to cause viremia in vertebrate hosts, including mammals. This raises the prospect that increased Ub activity may be an adaptive mechanism allowing these nairoviruses to infect a wider host range. While ticks are known to possess RNAi and Toll sensing-mediated antiviral responses, there is little information pertaining to whether Ub plays a significant role in arthropod responses to viral infection [46–48]. Further characterization of Ub systems in arthropods will be needed to shed light into these questions and would clarify the significance of vOTU enzymatic diversity in nairovirus adaptation for arthropod versus vertebrate hosts.

### Nairovirus vOTUs have activity towards poly-Ub linkages involved in significant immune signaling processes

In contrast to some mammalian DUBs, OTU proteases generally show poly-Ub linkage specificity that ranges from moderate to highly specific [38]. Nairovirus vOTUs reflect this tendency, possessing activity towards poly-Ub linkages that is neither highly promiscuous nor completely selective for a single linkage type with each vOTU possessesing its own respective preferences for the different linkages. While showing individual variation, the vOTUs consistently show the ability to process K6, K11, K48, and K63 linked di-Ub. The fact that nairovirus vOTUs generally show activity towards K48 and K63 di-Ub is significant. These well-studied forms of di-Ub have clearly established roles for cellular processes in general, as well as in antiviral responses specifically. It can be easily envisioned how disruption of K48 and K63-mediated functions could dampen antiviral responses. It is intriguing, however, that vOTUs as a whole also possess substantial activity towards K6 and K11 linkages. These forms of Ub have been studied much less extensively, with roles that have typically been associated with DNA damage responses and cell cycle regulation [49, 50]. As part of the L protein, vOTU activity would be restricted to the cytosol, raising questions as to whether these observed activities of vOTUs are incidental, or if there are important cytosolic functions of K6 and K11 linkages that could be manipulated. Recent studies have begun to expand knowledge of these linkages. Specifically, K11 poly-Ub has been associated with TNF signaling, providing a direct link to the innate immune response [51]. Even more recently, K6 has emerged as a key component in regulating mitophagy [52, 53]. Given the key role of mitochondria in innate immunity, this raises an interesting question of how vOTU activity could impact this process, and whether such manipulation could provide benefits for the virus [54]. What other functions remain to be identified for these linkages is still an open question, as well as how vOTUs may engage with them to modify cellular responses. The differences in linkage preferences between vOTUs implies potential differences in the degree to which specific viruses may influence these pathways. Alternatively, it’s possible that the relative importance of the linkages may differ in different hosts, and that different vOTU preferences reflects virus adaptation to their specific preferred hosts.

### Influence of genomic diversity on the nairovirus vOTU fold

The new vOTU structures reveal an array of conserved and divergent features. The conserved elements of Nairovirus vOTU structure distinguishes these from other OTU proteases, as highlighted by how they cluster together in a structure-based phylogenetic tree (Fig 1A, inset; [34, 55, 56]). Most notably, this includes the presence of two additional beta sheets and a helix at the N-terminus of vOTUs that are absent from eukaryotic OTUs. While possessing these characteristic features of the vOTU fold, the nairoviruses show distinguishable differences from each other that can be traced to specific residue differences. This is particularly noteworthy when looking at the relationship between the selectivity pocket and the observed Ub activity by a given protease. It is significant that vOTUs possessing the highest activity for Ub all possess highly hydrophobic residues in this region. While many of the vOTUs possessing robust DUB activity are closely related phylogenetically, the presence of substantial activity in the more distantly related QYBV vOTU demonstrates that it is not exclusive to this subset of viruses. This suggests the vOTU fold to be a flexible platform that has allowed DUB activity to evolve independently to the benefit of each virus. Beyond the central role of this pocket that is deep in the binding interface, the vOTUs also display structural diversity in more peripheral regions. This includes areas that have been observed to influence substrate binding in vOTUs, such as the α3 selectivity helix, suggesting a potential impact on how vOTUs engage with other proteins.

### Possible functions of the structural motif in the *Hughes orthonairovirus* species

Viruses in the *Hughes orthonairovirus* species possess a motif previously unobserved for OTU domains. This raises the question of whether this structural feature could have a functional impact. In particular, whether this motif could impact engagement with substrate. The effect of removing this motif from the FARV vOTU on Ub-AMC activity suggests that it can at least contribute to mono-Ub binding. This is consistent with what is observed when comparing FARV vOTU to a Ub-bound structure of CCHFV vOTU, where elements of this motif are in proximity for potential interactions with Ub (Fig 7A). This additional interface provided by the motif likely compensates for the presence of other less optimal factors for Ub binding, including an arginine that hinders interaction with the tail of Ub. Overall, these structural features suggest a mixture of elements that either promote or hinder interaction, with some that may carry a more dominant effect. The involvement of the structural motif in FARV vOTU formed by the two helices and intervening loop, which we also refer to as a substrate interacting bundle (SIB), suggests it may form a region that introduces potential to engage with otherwise inaccessible surfaces.

Interestingly, removing the SIB motif from FARV vOTU appears to have a lesser impact on di-Ub activity compared to mono-Ub (Fig 7B). This could be accounted for by the presence of an additional site of interaction in FARV vOTU that interacts with the proximal Ub. The existence of one or more subsites has been postulated as a mechanism for discriminating different di-Ub linkages based on the proximal Ub, and has been demonstrated in several mammalian OTUs [38, 39]. While not definitively observed in vOTUs, the ability to distinguish between different linkages implies a similar mechanism. The FARV vOTU mutants provide the first reported direct evidence identifying such a site in a vOTU, confirming that vOTUs can utilize this mechanism to distinguish various linkages. In addition to supplying potential leads for elucidating such sites in other vOTUs, it also demonstrates a case where this site can have a major impact on activity towards substrate, even when factors hindering binding with the distal Ub are present.

Although di-Ub wouldn’t directly interact with the SIB motif in a manner that would directly influence cleavage, it is possible that a more complex poly-Ub structure could engage with it. Modeling how tri-Ub might bind suggests that a K48 linkage could place one of the Ub molecules in close proximity to the structural motif (Fig 7C). In contrast, for the other linkages FARV vOTU most readily cleaves—K6, K11, and K63—this Ub would likely be too distant to form any interaction. Surprisingly, removal of the SIB motif has no noticeable impact on K48 tri-Ub cleavage, despite the apparent proximity the tri-Ub could have. It’s possible that tri-Ub may not possess a large enough architecture to be influenced by the SIB motif, and that a longer poly-Ub may interact with it. Additionally, Ub is able to form complex chains consisting of multiple linkage types [57]. It may be that the SIB motif can engage more effectively with these “heterotypic” Ub chains. Alternatively, the primary role of the SIB motif may go beyond Ub and facilitate interactions with other binding partners. The vOTU domain exists in the context of the multifunctional L protein. Apart from the vOTU domain, the structural features and dynamics of the nairovirus L protein are currently unknown. This leaves open the possibility that the SIB motif could be involved in binding another feature of the L protein to stabilize the overall architecture, or in facilitating interactions with other proteins. In addition, the SIB motif could potentially bind to other host factors in the innate immune system. Viruses in the *Hughes orthonairovirus* species have been isolated from birds or from ticks that infest them. The immune system of birds, including antiviral responses, possesses considerable differences from mammals in terms of what elements are present and how they are regulated (reviewed in [58] and [59]). This includes the apparent absence of an ISG15 homologue in birds. These differences from mammals raises the possibility that the SIB motif could play a role in adaptation to the avian innate immune system, perhaps by facilitating interactions with proteins other than Ub. In addition, the lack of ISG15 in birds leaves open the possibility that the motif could engage with other Ub-like entities that are involved in regulating the innate immune response.

### Challenges to defining vOTU function

While divergent vOTUs possess the ability to engage with Ub, it is possible that this may not be the only, or even predominant function of all vOTUs. In the case of ERVEV, it has been observed that it possesses poor activity towards Ub, while showing potent ability to engage with ISG15 (Fig 2; [32, 33]). This raises the possibility that other vOTUs that possess poor Ub activity may be able to engage with other Ub-like entities. While none of the new vOTUs assessed possess notable deISGylase activity, the availability of AMC-derived substrates is limited to human ISG15 (hISG15). In contrast to Ub, ISG15 shows considerable species-species variances that have been shown to impact binding with viral proteins, including vOTUs from nairoviruses [33, 60, 61]. This leaves open the possibility that vOTUs, while not engaging with hISG15, may still possess the ability to interact with ISG15 from species they productively infect. The presence of arginine, lysine, or glutamine in the selectivity pocket of several of the vOTUs, while not ideal for Ub, may still allow them to engage with other substrates. The structure of the ERVEV vOTU bound to mouse ISG15 (mISG15) has a gap in the area that Ub’s Leu8 would typically occupy (Fig 5B, Panel I). Modeling suggests that this feature would also be more permissive of binding with vOTUs possessing a bulky residue such as arginine at position 131 (Fig 5B, Panel II). This gap is caused by a pairing of Glu87 with Lys148 in mISG15 that pulls the sidechain of Glu87 away from the interface, and suggests a possible mechanism that could allow vOTUs with hydrophilic or bulky residues to effectively engage with non-Ub moieties. As highlighted by the lack of ISG15 in birds, however, it’s also possible that vOTUs, particularly in the *Hughes orthonairovirus* species, may engage with other Ub-like entities that can modulate the immune response. The lack of either Ub or ISG15 activity in a number of vOTUs further accentuates this possibility, implying possible biochemical functions that have yet to be characterized among vOTUs. Further developments shedding light on these questions could yield key insights into these influential virus-host interactions.

### Conclusion

The recent increase in genomic characterization of nairoviruses has uncovered a wealth of diversity among them. While our knowledge of nairoviral sequence diversity has expanded, much is still unknown on how this variability affects virus-host relationships. The exact range of vertebrate hosts and their disease state upon infection is not presently known for all members of the *Nairoviridae* family. This novel characterization of nairovirus vOTUs reveals a diversity in the ability to engage mono- and poly-Ub that mirrors the genomic diversity. Additionally, this study uncovers motifs that appear to play a predominant role in determining these preferences, making it feasible to begin predicting DUB activity in uncharacterized or newly discovered nairoviruses. Given the presence of robust DUB activity in nairoviruses known to infect humans, including CCHFV and NSDV, this could serve as an early flag for assessing the risk posed by emerging viruses, and may shed light on the evolutionary trends leading to some viruses to having this capability over others. Further, these new structure and activity insights provide a platform to continue the development of robust tools, such as poly-Ub specific vOTUs, that can be paired with reverse genetics systems to better understand the role of the vOTU in the course of a viral infection and how differences in certain activities impact nairoviruses. Such knowledge could help propel the field in fully elucidating the detailed functional mechanism of the vOTU in the viral life cycle, potentially aiding in the development of better disease model systems. In addition, it provides insight that will further gauge the prospects of the vOTU as a therapeutic target for nairovirus-caused diseases such as CCHF, either through the development of specific inhibitors or live attenuated virus vaccines. Further, the diversity of the vOTU suggests a potential relationship with viral host adaptation, and that the role of the vOTU may extend beyond its well-known function in engaging with Ub and/or ISG15.

## Methods

### Construction, expression, and purification of vOTUs

The vOTUS were constructed and expressed as previously described in published methods [32, 55]. Purification of QYBV, TAGV, and DGKV were carried out as previously described. For FARV, a slightly different approach altered from the previously described method was used to optimize the expression. *E*. *coli* strains with vOTUs from FARV were grown at 37°C in 6 L of Luria-Bertani broth with 100 μg/ml ampicillin. Once the optical density reached 0.6–0.8, 0.8 mM isopropyl-β-D-thiogalactopyranoside (IPTG) was added to induce gene expression. The temperature was then dropped to 25°C and expression continued overnight. The culture was subsequently centrifuged at 5000xg for 10 minutes and the pelleted cells stored at -80°C.

### Enzymatic assays

Assays were carried out as described previously [32, 33]. Briefly, assays were run in 100 mM NaCl, 50 mM HEPES [pH 7.5], 0.01 mg/mL BSA, 5 mM dithiothreitol (DTT) at 25°C. Reactions were run in 96-well plates with a 50 μl reaction volume using a CLARIOstar plate reader (BMG Labtech, Inc.). For Ub-AMC, all vOTUs were assessed at a final enzyme concentration of 4 nM. For ISG15-AMC, vOTUs were assessed at a final enzyme concentration of 20 nM with the exception of NSDV, GANV, and ERVEV, which were run at a final enzyme concentration of 4 nM due to the high activity towards the substrate. Both Ub-AMC and ISG15-AMC assays were run at a final substrate concentration of 1 μM. Assays with Ub-Rh110 were run under the same reaction conditions as Ub-AMC with instrument settings adjusted to optimize detection of the fluorophore. For DGKV vOTU additional assays were run with the WT and E152D mutant using the peptide Z-RLRGG-AMC (Bachem) substrate with protease concentrations of 4 μM and a substrate concentration of 50 μM.

### Poly-Ub cleavage assays

Assays with FRET TAMRA/QXL pair tagged K11, K48, and K63 di-Ub substrates were performed as previously described with 4 nM vOTU and 1 μM substrate [32]. Untagged poly-Ub cleavage assays were adapted from the previously published method. Briefly, 4 nM of each vOTU was tested against 10 μM Linear (M1), K6, K11, K27, K29, K33, K48, and K63 linked di-Ub (Boston Biochem, MA). Reactions were initiated by the addition of vOTU and incubated at 37°C in reaction buffer (100 mM NaCl, 5 mM HEPES [pH 7.5], 2 mM DTT). The reactions were stopped at the time points indicated by mixing 5 μl of each reaction with 2x Laemmli sample buffer and heat killed by boiling at 98°C for 5 minutes. The cleavage over time was visualized using 8–16% Mini-Protean TGX precast gels (Bio-Rad) by Coomassie staining. Assays with K48 and K63 linked tri-Ub were run in the same manner except that tri-Ub was present at 20 μM.

### Crystallization of vOTUs

All four vOTUs were screened against a series of Qiagen NeXtal suites in a 96-well hanging drop format with a TTP LabTech Mosquito (TTP Labtech, Herfordshire, United Kingdom). QYBV vOTU was screened at 11.36 mg/ml, TAGV vOTU at 12.70 mg/ml, DGKV vOTU at 10.96 mg/ml, and FARV vOTU at 10.96 mg/ml. Initial hits were optimized along salt, precipitant, and pH gradients as applicable. The TAGV and FARV vOTU hits were also optimized with an Additive HT Screen from Hampton Research. Final optimized crystals for all four vOTUs were flash frozen in cryoprotective solutions. For QYBV vOTU, the final optimized crystals were in 0.3 M magnesium acetate and 16% PEG 3350, with 0.3 M magnesium acetate, 20% PEG 3350, and 18% of a 1:1:1 solution of ethylene glycol, dimethyl sulfoxide, and glycerol (EDG) as the cryoprotectant. The final crystals for TAGV vOTU were grown in 0.15 M magnesium formate, 22% PEG 3350, with 0.25 M TCEP as an additive, with a cryoprotectant solution consisting of 0.15 M magnesium formate, 22% PEG 3350, 18% EDG. Final optimized crystals for DGKV vOTU were found in the condition with 0.1 M citric acid pH 3.5, 13% PEG 6000 and were flash frozen in 0.1 M citric acid pH 3.5, 20% PEG 6000, 18% EDG. For FARV vOTU, the final optimized crystals were grown in 0.3 M magnesium chloride, 0.1 M MES pH 6.5, and 8% PEG 4000, and flash frozen in 0.3 M magnesium chloride, 0.1 M MES pH 6.5 and 20% PEG 4000 as the cryoprotectant. For selenomethionyl (Se-Met) derivative QYBV vOTU crystals, bacterial cells were grown in minimal media to OD 0.6 and induced with 0.8 mM IPTG at 37°C for 4 hrs. Prior to induction, the cultures were supplemented with eight amino acids (Leu, Ile, Val, and Trp at 0.05 g/L; Thr, Lys, Phe, and Cys at 0.1 g/L) as well as selenomethionine (0.12 g/L). Cells were harvested and protein purified as previously described. Final crystals were grown in 0.3 M magnesium acetate, 16% PEG 3350, in drops formed from 1 μl of solution and 2 ul of 9.45 mg/ml protein. Native datasets of the QYBV, DGKV, TAGV, and FARV vOTUs were collected at a wavelength of 1 Å. A Se-Met single anomalous dispersion (SAD) dataset for QYBV vOTU was collected at the absorption edge of Se at 0.9792 Å.

### Structural solutions of vOTUs

The data sets were indexed, integrated and scaled with HKL-2000 [62]. Experimental phasing of the Se-Met-SAD dataset was performed using the Phenix suite of programs [63]. HySS was utilized to locate the Se-Met sites, with Phaser solving the experimental phases [64–66]. Initial model building was performed using AutoBuild, with subsequent cycles of Refinement and model building carried out in Phenix and Coot ([63, 67, 68]. This structure was then used as a search model to solve the QYBV vOTU native dataset by Molecular Replacement in Phaser [66]. The other vOTUs were solved by Molecular Replacement. A QYBV vOTU-based homology model was used to solve DGKV vOTU, while homology models based on DUGV vOTU (PDB entry 4HXD) were used to solve TAGV vOTU and FARV vOTU. All the structures were built with Autobuild, followed by alternating manual building and refinement in Coot and Phenix. Structures were validated using the MolProbity server [69].

### Generation of vOTU and Ub mutants

Mutations were made using the QuikChange Lightning Kit according to the manufacturer’s protocol (Agilent Technologies, Inc.). The PCR-generated plasmids were transformed into Escherichia coli NEB-5α cells by heat shock. The mutant plasmids were confirmed by sequencing and transformed into T7 Express cells (New England Biolabs).

### Isothermal Titration Calorimetry of TAGV vOTU binding with Ub, Ub-L8A, and Ub-L8N

T7 Express cells expressing Ub, Ub-L8A, and Ub-L8N in pET-15b were grown to OD 0.6–0.8 at 37°C. Expression was induced with 0.5 mM IPTG and continued at 18°C overnight. The cells were pelleted and stored as described above. The pellet was resuspended in 500 mM NaCl, 50 mM Tris [pH 7.5] supplemented with lysozyme at 4°C for 30 minutes. The cells were sonicated on ice at 70% power with a 50% duty cycle for a total of 6 minutes, followed centrifugation at 48,000xg for 45 minutes. The supernatant was filtered through a 0.8 μm and applied to a gravity flow Ni-NTA column (GoldBio) pre-equilibrated with 500 mM NaCl, 50 mM Tris [pH 7.5]. The column was washed with the same buffer containing 30 mM imidazole, followed by elution with 300 mM imidazole. Thrombin was added to cleave the 6X His-tag and the elution dialyzed overnight in 250 mM NaCl, 25 mM HEPES [pH 7.5], 2 mM DTT at 4°C. After dialysis the protein was filtered through a 0.22 μm membrane and run over a Superdex 200 column (GE Healthcare) equilibrated with 100 mM NaCl, 5 mM HEPES [pH 7.5], 2 mM DTT. The fractions were pooled based on the chromatogram and concentrated to ~2–2.5 mM, supplemented with 5% glycerol, and flash frozen in liquid nitrogen followed by storage at -80°C until further use. TAGV vOTU was purified as previously described and dialyzed alongside Ub and Ub-L8N in 150 mM NaCl, 50 mM HEPES [pH 7.5], 1 mM TCEP overnight at 4°C. ITC was performed with a Microcal PEAQ-ITC (Malvern, Worcestershire, UK). Ub or Ub-L8N were titrated into the cell in series of 19 injections, 2 μL each with a spacing of 180 seconds. The temperature was kept constant at 25°C with a reference power ranging from 6–10 μcal/s. For the TAGV vOTU binding with WT Ub the vOTU was present in the cell at 114–134 μM with Ub at 1.26–1.29 mM in the syringe. For TAGV vOTU binding with Ub-L8A the vOTU was present in the cell at 111–114 μM with Ub-L8A at 1.32–1.35 mM in the syringe. For TAGV vOTU binding with Ub-L8N the vOTU was present in the cell at 234–235 μM in the cell and Ub-L8N at 4.67–4.74 mM in the syringe. The data was processed in the Microcal PEAQ-ITC Analysis Software and fit to an independent model. Values for Ub and Ub-L8N represent the average and standard deviation of three independent runs for each experiment.

### Accession numbers

Final protein structures were deposited in the Protein Data Bank with IDs 6DWX, 6DX1, 6DX2, 6DX3, and 6DX5 for Se-Met QYBV vOTU, native QYBV vOTU, DGKV vOTU, TAGV vOTU, and FARV vOTU respectively.

## Supporting information

S1 Table Viruses and sequence accession numbers. (DOCX)

S1 Fig Comparison of commercially available di-UB FRET TAMRA/QXL substrates. Activities of the vOTUs towards the different donor-quencher pair positions of K48 and K63 di-Ub FRET substrates. Values shown are the mean ± standard deviation of two independent experiments.
(TIF)

S2 Fig Global structural comparison of vOTUs. (A) Overall structures of the TAGV, DGKV, and QYBV vOTUs with those of the previously solved CCHFV (PDB ID 3PRP), DUGV (PDB ID 4HXD), and ERVEV (PDB ID 5JZE) vOTUs. (B) Histogram of root mean square deviation of vOTU alpha carbons when measured against CCHFV vOTU. General regions highlighted in the text are indicated by red brackets.
(TIF)

S3 Fig ITC Isotherms of TAGV vOTU-Ub binding and biochemical environment of FARV vOTU Leu13. (A) Raw heat and integrated curves where binding occurs for representative runs of TAGV vOTU binding with Ub, Ub-L8A, and Ub-L8N. (B) Closeup of Leu13 and the surrounding hydrophobic region.
(TIF)

S1 File Validation report for SeMet-phased QYBV vOTU. (PDF)

S2 File Validation report for native QYBV vOTU. (PDF)

S3 File Validation report for DGKV vOTU. (PDF)

S4 File Validation report for TAGV vOTU. (PDF)

S5 File Validation report for FARV vOTU. (PDF)
