# Supplementary material for: Probing the impact of nairovirus genomic diversity on viral ovarian tumor domain protease (vOTU) structure and deubiquitinase activity
Source: PLoS Pathog. 2019 Jan 10;15(1):e1007515. doi: 10.1371/journal.ppat.1007515 (PMC6343935; doi:10.1371/journal.ppat.1007515)
Supplement: S1 Table — (DOCX) [file ppat.1007515.s001.docx]

**S1 Table. Viruses and sequence accession numbers**

| **Name** | **Abbreviation** | **Species** | **Accession number** |
| --- | --- | --- | --- |
| Crimean-Congo hemorrhagic fever virus | CCHFV | *Crimean-Congo hemorrhagic fever orthonairovirus* | AAQ98866.2 |
| Ganjam virus | GANV | *Nairobi sheep disease orthonairovirus* | AMT75401.1 |
| Nairobi sheep disease virus | NSDV |  | ACH99799.1 |
| Dugbe virus | DUGV | *Dugbe orthonairovirus* | AAB18834.1 |
| Kupe virus | KUPEV |  | ABY82502.1 |
| Hazara virus | HAZV | *Hazara orthonairovirus* | AAZ38668.1 |
| Tofla virus | TFLV |  | YP_009227122.1 |
| Taggert virus | TAGV | *Sakhalin orthonairovirus* | AMT75428.1 |
| Tillamook virus | TILLV |  | AMT75431.1 |
| Paramushir virus | PRMV |  | AKC89337.1 |
| Avalon virus | AVAV |  | AMT75377.1 |
| Artashat virus | ARTSV | *Artashat orthonairovirus* | AKC89352.1 |
| Thiafora virus | TFAV | *Thiafora orthonairovirus* | ALD84355.1 |
| Erve virus | ERVEV |  | AFH89032.1 |
| Hughes virus | HUGV | *Hughes orthonairovirus* | AKC89316.1 |
| Farallon virus | FARV |  | AMT75398.1 |
| Raza virus | RAZAV |  | AMT75416.1 |
| Punta Salinas virus | PSV |  | AMT75410.1 |
| Zirqa virus | ZIRV |  | AMT75437.1 |
| Soldado virus | SOLV |  | AMT75425.1 |
| Great Saltee virus | GRSV |  | AMT75404.1 |
| Caspiy virus | CASV |  | AKC89346.1 |
| Abu Hammad virus | AHV | *Dera Ghazi Khan orthonairovirus* | AMT75434.1 |
| Dera Ghazi Khan virus | DGKV |  | AMT75389.1 |
| Sapphire II virus | SAPV |  | AMT75422.1 |
| Wēnzhōu tick virus | WzTV | *Tamdy orthonairovirus* | YP_009304993.1 |
| Burana virus | BURV |  | AKC89349.1 |
| Huángpí tick virus 1 | HpTV-1 |  | YP_009293587.1 |
| Tǎchéng tick virus 1 | TcTV-1 |  | YP_009304986.1 |
| Tamdy virus | TDYV |  | AKC89328.1 |
| Yogue virus | YOGV | *Kasokero orthonairovirus* | YP_009246486.1 |
| Leopards Hill virus | LPHV |  | BAP90971.1 |
| Qalyub virus | QYBV | *Qalyub orthonairovirus* | AKC89319.1 |
| Geran virus | GERV |  | AKC89340.1 |
| Chim virus | CHIMV | *Chim orthonairovirus* | AKC89343.1 |
| Gossas virus | GOSV | *Keterah orthonairovirus* | ALD83626.1 |
| Issyk-kul virus | ISKV |  | AII79373.1 |
| Uzun Agach virus | UZAV |  | AKC89313.1 |
| Keterah virus | KTRV |  | YP_009361838.1 |
