# Supplementary figures and images for: Probing the impact of nairovirus genomic diversity on viral ovarian tumor domain protease (vOTU) structure and deubiquitinase activity
Source: PLoS Pathog. 2019 Jan 10;15(1):e1007515. doi: 10.1371/journal.ppat.1007515 (PMC6343935; doi:10.1371/journal.ppat.1007515)

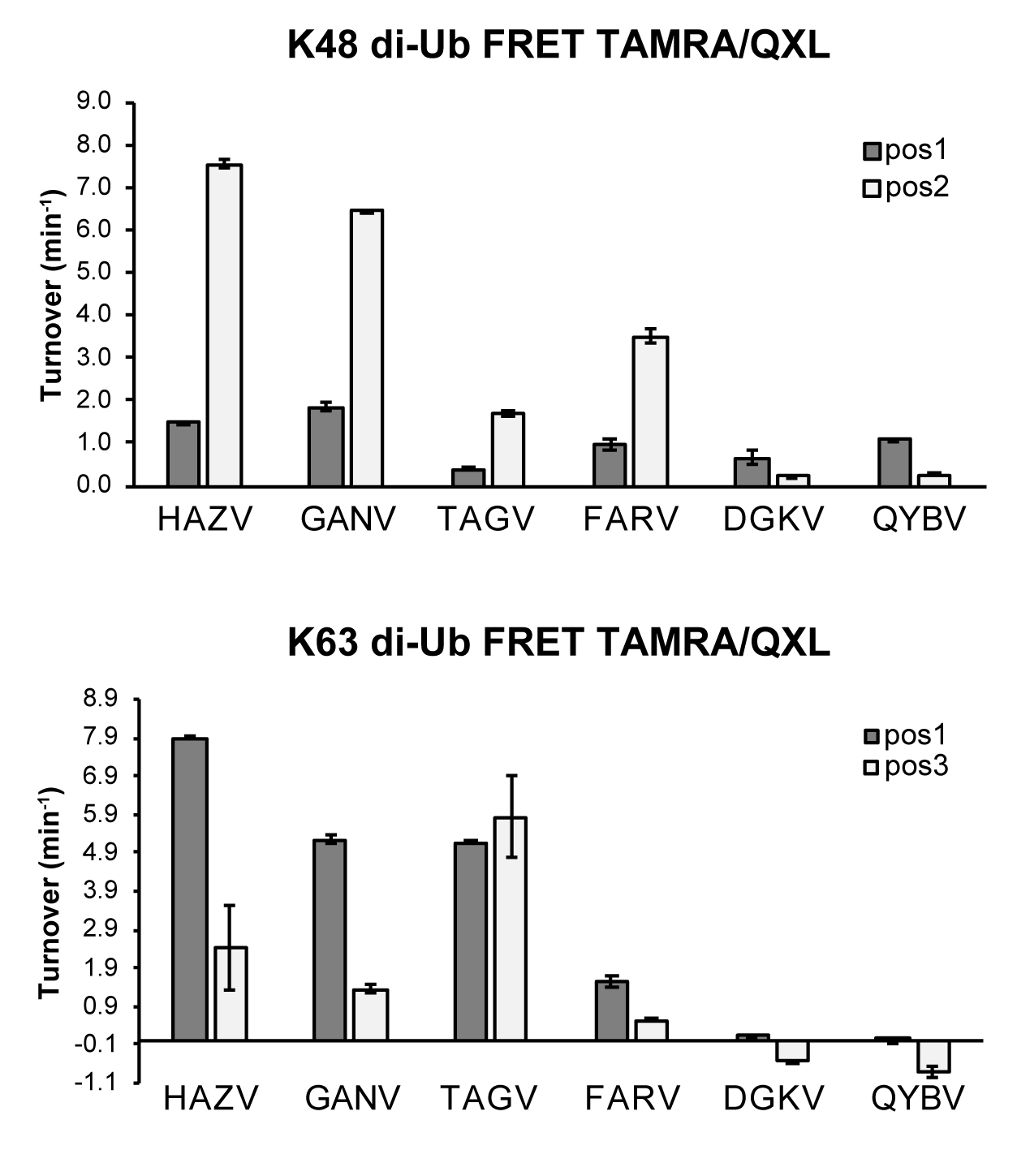

Supplement: S1 Fig — Activities of the vOTUs towards the different donor-quencher pair positions of K48 and K63 di-Ub FRET substrates. Values shown are the mean ± standard deviation of two independent experiments. (TIF) [file ppat.1007515.s002.tif]

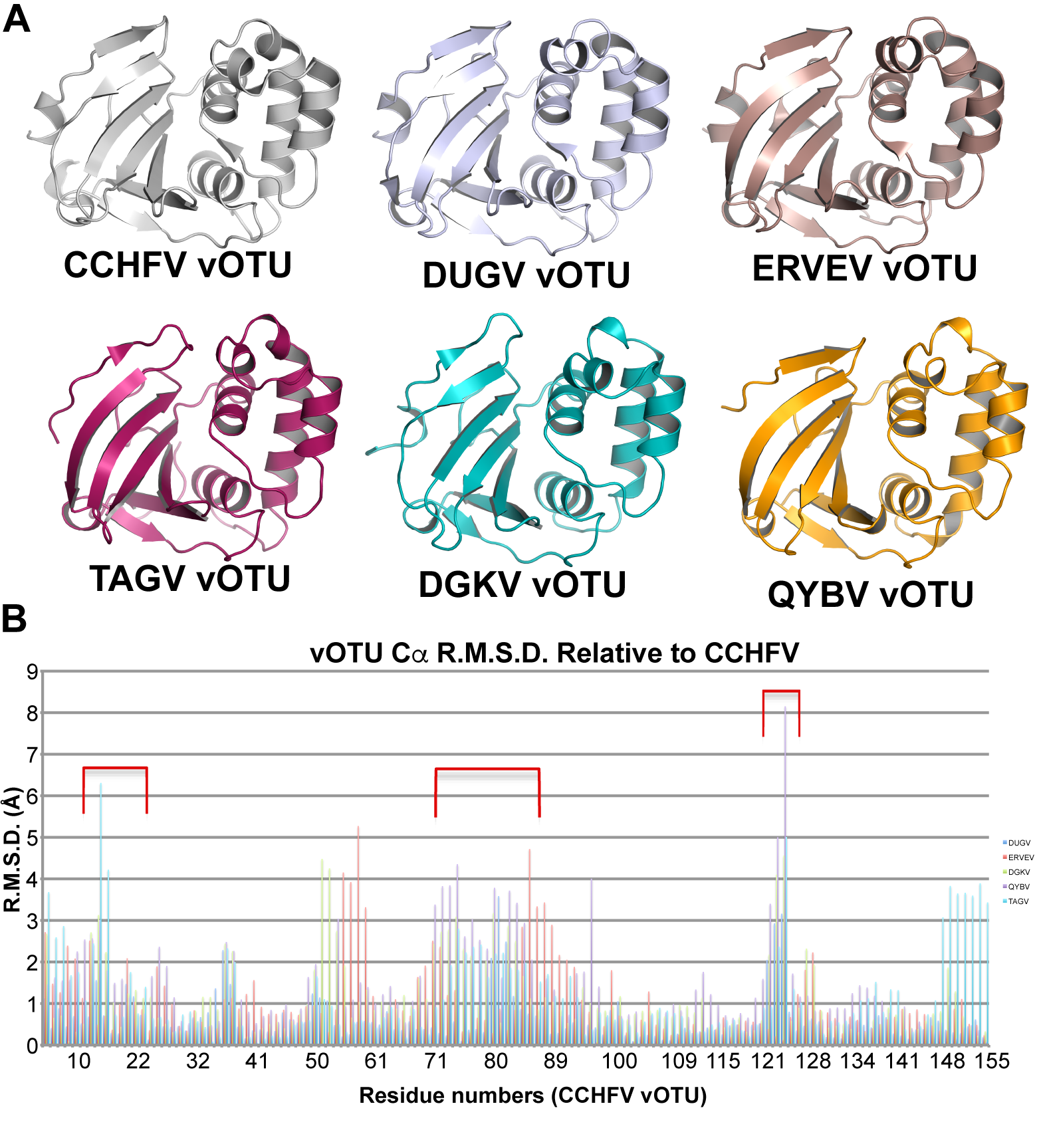

Supplement: S2 Fig — (A) Overall structures of the TAGV, DGKV, and QYBV vOTUs with those of the previously solved CCHFV (PDB ID 3PRP), DUGV (PDB ID 4HXD), and ERVEV (PDB ID 5JZE) vOTUs. (B) Histogram of root mean square deviation of vOTU alpha carbons when measured against CCHFV vOTU. General regions highlighted in the text are indicated by red brackets. (TIF) [file ppat.1007515.s003.tif]

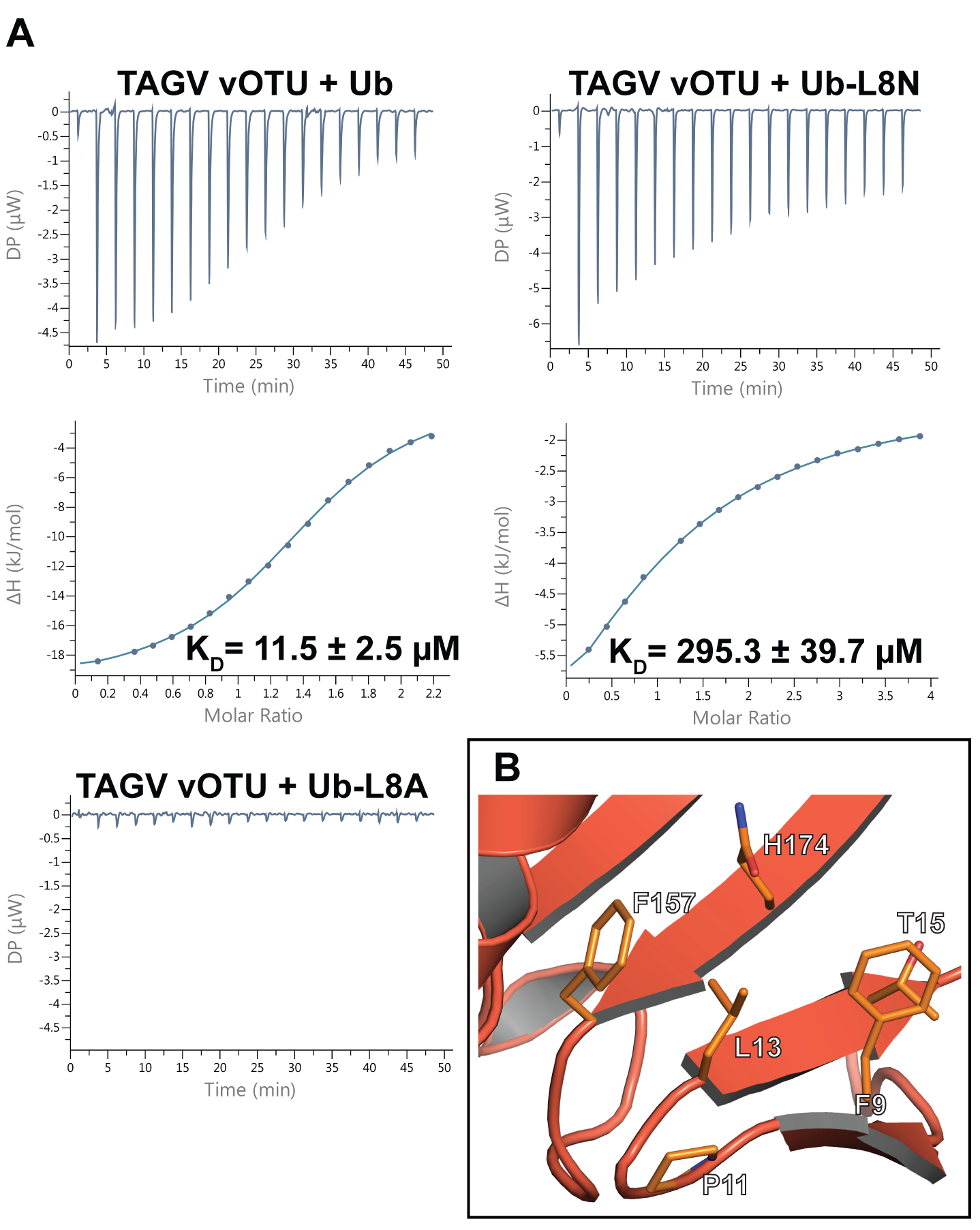

Supplement: S3 Fig — (A) Raw heat and integrated curves where binding occurs for representative runs of TAGV vOTU binding with Ub, Ub-L8A, and Ub-L8N. (B) Closeup of Leu13 and the surrounding hydrophobic region. (TIF) [file ppat.1007515.s004.tif]
