# Supplementary material for: Probing the impact of nairovirus genomic diversity on viral ovarian tumor domain protease (vOTU) structure and deubiquitinase activity
Source: PLoS Pathog. 2019 Jan 10;15(1):e1007515. doi: 10.1371/journal.ppat.1007515 (PMC6343935; doi:10.1371/journal.ppat.1007515)
Supplement: S2 File — (PDF) [file ppat.1007515.s006.pdf]

Full wwPDB X-ray Structure Validation Report (i)

Jul 2, 2018 – 11:23 AM EDT

PDB ID	:	6DX1
Title	:	Crystal structure of the viral OTU domain protease from Qalyub virus
Deposited on	:	2018-06-28
Resolution	:	1.65 Å(reported)

This is a Full wwPDB X-ray Structure Validation Report.

This report is produced by the wwPDB biocuration pipeline after annotation of the structure.

We welcome your comments at *validation@mail.wwpdb.org*
A user guide is available at
https://www.wwpdb.org/validation/2017/XrayValidationReportHelp
with specific help available everywhere you see the (i) symbol.

CONFIDENTIAL VALIDATION REPORT

The following versions of software and data (see references (i)) were used in the production of this report:

MolProbity	:	4.02b-467
Xtriage (Phenix)	:	1.13
EDS	:	rb-20031172
Percentile statistics	:	20171227.v01 (using entries in the PDB archive December 27th 2017)
Refmac	:	5.8.0158
CCP4	:	7.0 (Gargrove)
Ideal geometry (proteins)	:	Engh & Huber (2001)
Ideal geometry (DNA, RNA)	:	Parkinson et al. (1996)
Validation Pipeline (wwPDB-VP)	:	rb-20031172

1 Overall quality at a glance (i)

The following experimental techniques were used to determine the structure:
X-RAY DIFFRACTION

The reported resolution of this entry is 1.65 Å.

Percentile scores (ranging between 0-100) for global validation metrics of the entry are shown in the following graphic. The table shows the number of entries on which the scores are based.

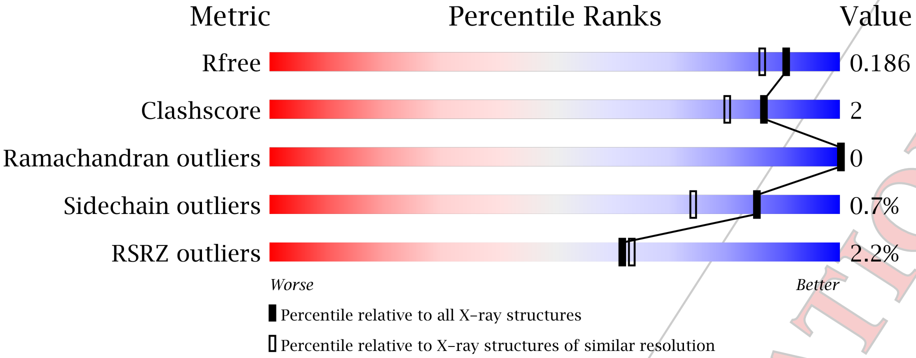

Metric	Whole archive (#Entries)	Similar resolution (#Entries, resolution range(Å))
R_{free}	111664	1521 (1.66-1.66)
Clashscore	122126	1616 (1.66-1.66)
Ramachandran outliers	120053	1584 (1.66-1.66)
Sidechain outliers	120020	1584 (1.66-1.66)
RSRZ outliers	108989	1487 (1.66-1.66)

The table below summarises the geometric issues observed across the polymeric chains and their fit to the electron density. The red, orange, yellow and green segments on the lower bar indicate the fraction of residues that contain outliers for >=3, 2, 1 and 0 types of geometric quality criteria. A grey segment represents the fraction of residues that are not modelled. The numeric value for each fraction is indicated below the corresponding segment, with a dot representing fractions <=5% The upper red bar (where present) indicates the fraction of residues that have poor fit to the electron density. The numeric value is given above the bar.

Mol	Chain	Length	Quality of chain
1	A	174	.% / 85% • 13%
1	B	174	2% / 84% 5% 11%
1	C	174	3% / 83% • 13%

CONFIDENTIAL VALIDATION REPORT

2 Entry composition (i)

There are 2 unique types of molecules in this entry. The entry contains 4339 atoms, of which 0 are hydrogens and 0 are deuteriums.

In the tables below, the ZeroOcc column contains the number of atoms modelled with zero occupancy, the AltConf column contains the number of residues with at least one atom in alternate conformation and the Trace column contains the number of residues modelled with at most 2 atoms.

- Molecule 1 is a protein called RNA-dependent RNA polymerase.

Mol	Chain	Residues	Atoms					ZeroOcc	AltConf	Trace
1	B	155	Total 1303	C 845	N 214	O 239	S 5	0	0	0
1	A	151	Total 1269	C 824	N 208	O 232	S 5	0	0	0
1	C	151	Total 1269	C 824	N 208	O 232	S 5	0	0	0

There are 27 discrepancies between the modelled and reference sequences:

Chain	Residue	Modelled	Actual	Comment	Reference
B	165	SER	-	expression tag	UNP A0A191KWB3
B	166	GLY	-	expression tag	UNP A0A191KWB3
B	167	SER	-	expression tag	UNP A0A191KWB3
B	168	HIS	-	expression tag	UNP A0A191KWB3
B	169	HIS	-	expression tag	UNP A0A191KWB3
B	170	HIS	-	expression tag	UNP A0A191KWB3
B	171	HIS	-	expression tag	UNP A0A191KWB3
B	172	HIS	-	expression tag	UNP A0A191KWB3
B	173	HIS	-	expression tag	UNP A0A191KWB3
A	165	SER	-	expression tag	UNP A0A191KWB3
A	166	GLY	-	expression tag	UNP A0A191KWB3
A	167	SER	-	expression tag	UNP A0A191KWB3
A	168	HIS	-	expression tag	UNP A0A191KWB3
A	169	HIS	-	expression tag	UNP A0A191KWB3
A	170	HIS	-	expression tag	UNP A0A191KWB3
A	171	HIS	-	expression tag	UNP A0A191KWB3
A	172	HIS	-	expression tag	UNP A0A191KWB3
A	173	HIS	-	expression tag	UNP A0A191KWB3
C	165	SER	-	expression tag	UNP A0A191KWB3
C	166	GLY	-	expression tag	UNP A0A191KWB3
C	167	SER	-	expression tag	UNP A0A191KWB3
C	168	HIS	-	expression tag	UNP A0A191KWB3
C	169	HIS	-	expression tag	UNP A0A191KWB3

Continued on next page...

CONFIDENTIAL VALIDATION REPORT

Continued from previous page...

Chain	Residue	Modelled	Actual	Comment	Reference
C	170	HIS	-	expression tag	UNP A0A191KWB3
C	171	HIS	-	expression tag	UNP A0A191KWB3
C	172	HIS	-	expression tag	UNP A0A191KWB3
C	173	HIS	-	expression tag	UNP A0A191KWB3

- Molecule 2 is water.

Mol	Chain	Residues	Atoms	ZeroOcc	AltConf
2	B	167	Total O<br>167 167	0	0
2	A	180	Total O<br>180 180	0	0
2	C	151	Total O<br>151 151	0	0

CONFIDENTIAL VALIDATION REPORT

3 Residue-property plots (i)

These plots are drawn for all protein, RNA and DNA chains in the entry. The first graphic for a chain summarises the proportions of the various outlier classes displayed in the second graphic. The second graphic shows the sequence view annotated by issues in geometryand electron density. Residues are color-coded according to the number of geometric quality criteria for which they contain at least one outlier: green = 0, yellow = 1, orange = 2 and red = 3 or more. A red dot above a residue indicates a poor fit to the electron density (RSRZ > 2). Stretches of 2 or more consecutive residues without any outlier are shown as a green connector. Residues present in the sample, but not in the model, are shown in grey.

- Molecule 1: RNA-dependent RNA polymerase

Chain B:

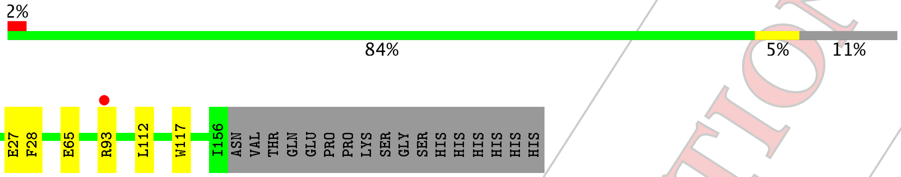

- Molecule 1: RNA-dependent RNA polymerase

Chain A:

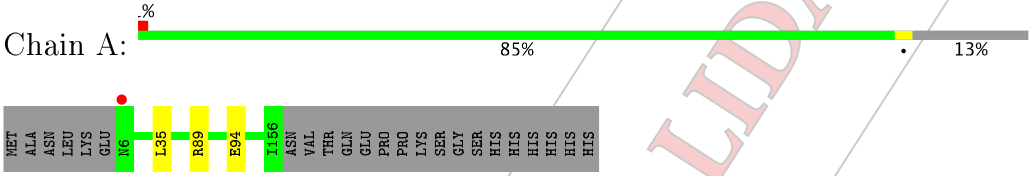

- Molecule 1: RNA-dependent RNA polymerase

Chain C:

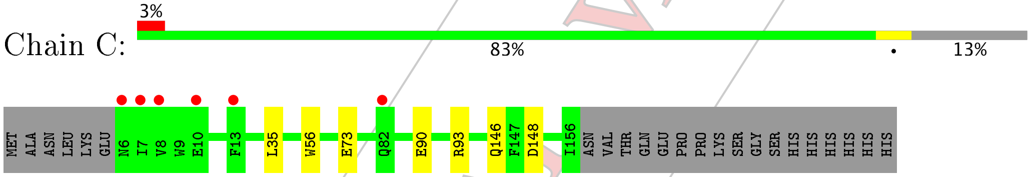

CONFIDENTIAL VALIDATION REPORT

4 Data and refinement statistics (i)

Property	Value	Source
Space group	P 32 2 1	Depositor
Cell constants<br>a, b, c, α, β, γ	110.47Å 110.47Å 106.23Å<br>90.00° 90.00° 120.00°	Depositor
Resolution (Å)	38.28 – 1.65<br>38.29 – 1.65	Depositor<br>EDS
% Data completeness<br>(in resolution range)	99.9 (38.28-1.65)<br>99.9 (38.29-1.65)	Depositor<br>EDS
R_{merge}	(Not available)	Depositor
R_{sym}	(Not available)	Depositor
$< I/\sigma(I) >$ [1]	1.85 (at 1.65Å)	Xtriage
Refinement program	PHENIX (1.11.1_2575: ???)	Depositor
R, R_{free}	0.171 , 0.186<br>0.171 , 0.186	Depositor<br>DCC
R_{free} test set	4570 reflections (5.09%)	wwPDB-VP
Wilson B-factor (Å^2)	22.2	Xtriage
Anisotropy	0.289	Xtriage
Bulk solvent k_{sol}(e/Å^3), B_{sol}(Å^2)	0.40 , 45.5	EDS
L-test for twinning[2]	$< \|L\| > = 0.49$, $< L^2 > = 0.33$	Xtriage
Estimated twinning fraction	0.021 for -h,-k,l	Xtriage
F_o,F_c correlation	0.97	EDS
Total number of atoms	4339	wwPDB-VP
Average B, all atoms (Å^2)	26.0	wwPDB-VP

Xtriage's analysis on translational NCS is as follows: *The largest off-origin peak in the Patterson function is 2.99% of the height of the origin peak. No significant pseudotranslation is detected.*

[1]Intensities estimated from amplitudes.

[2]Theoretical values of $< |L| >$, $< L^2 >$ for acentric reflections are 0.5, 0.333 respectively for untwinned datasets, and 0.375, 0.2 for perfectly twinned datasets.

CONFIDENTIAL VALIDATION REPORT

5 Model quality (i)

5.1 Standard geometry (i)

The Z score for a bond length (or angle) is the number of standard deviations the observed value is removed from the expected value. A bond length (or angle) with $|Z| > 5$ is considered an outlier worth inspection. RMSZ is the root-mean-square of all Z scores of the bond lengths (or angles).

Mol	Chain	Bond lengths		Bond angles	
		RMSZ	$\#\|Z\| >5$	RMSZ	$\#\|Z\| >5$
1	A	0.37	0/1310	0.52	0/1782
1	B	0.37	0/1344	0.54	0/1827
1	C	0.35	0/1310	0.50	0/1782
All	All	0.36	0/3964	0.52	0/5391

There are no bond length outliers.

There are no bond angle outliers.

There are no chirality outliers.

There are no planarity outliers.

5.2 Too-close contacts (i)

In the following table, the Non-H and H(model) columns list the number of non-hydrogen atoms and hydrogen atoms in the chain respectively. The H(added) column lists the number of hydrogen atoms added and optimized by MolProbity. The Clashes column lists the number of clashes within the asymmetric unit, whereas Symm-Clashes lists symmetry related clashes.

Mol	Chain	Non-H	H(model)	H(added)	Clashes	Symm-Clashes
1	A	1269	0	1178	2	0
1	B	1303	0	1214	7	0
1	C	1269	0	1178	9	0
2	A	180	0	0	1	2
2	B	167	0	0	4	2
2	C	151	0	0	5	1
All	All	4339	0	3570	17	3

The all-atom clashscore is defined as the number of clashes found per 1000 atoms (including hydrogen atoms). The all-atom clashscore for this structure is 2.

All (17) close contacts within the same asymmetric unit are listed below, sorted by their clash magnitude.

CONFIDENTIAL VALIDATION REPORT

Atom-1	Atom-2	Interatomic distance (Å)	Clash overlap (Å)
1:C:146:GLN:OE1	2:C:201:HOH:O	1.97	0.81
1:A:94:GLU:OE2	2:A:201:HOH:O	2.00	0.80
1:B:2:ASN:N	2:B:202:HOH:O	2.18	0.75
1:B:65:GLU:OE2	2:B:201:HOH:O	2.13	0.66
1:B:93:ARG:NH2	2:B:204:HOH:O	2.29	0.65
1:C:73:GLU:OE2	2:C:202:HOH:O	2.14	0.64
1:C:90:GLU:OE1	2:C:204:HOH:O	2.16	0.60
1:C:148:ASP:OD2	2:C:203:HOH:O	2.16	0.60
1:C:93:ARG:HG2	1:C:93:ARG:HH11	1.68	0.58
1:C:93:ARG:HD3	2:C:291:HOH:O	2.09	0.51
1:B:21:VAL:HG11	1:B:117:TRP:HZ3	1.76	0.51
1:A:35:LEU:HD11	1:C:35:LEU:HD21	1.94	0.48
1:B:65:GLU:OE2	2:B:203:HOH:O	2.20	0.48
1:B:93:ARG:HA	1:B:93:ARG:HD3	1.65	0.45
1:B:27:GLU:HG2	1:B:28:PHE:CE2	2.52	0.44
1:C:93:ARG:CG	1:C:93:ARG:HH11	2.32	0.43
1:C:90:GLU:O	1:C:93:ARG:HG3	2.19	0.42

All (3) symmetry-related close contacts are listed below. The label for Atom-2 includes the symmetry operator and encoded unit-cell translations to be applied.

Atom-1	Atom-2	Interatomic distance (Å)	Clash overlap (Å)
2:B:213:HOH:O	2:A:314:HOH:O[4_565]	1.98	0.22
2:A:358:HOH:O	2:A:371:HOH:O[3_445]	2.10	0.10
2:B:325:HOH:O	2:C:341:HOH:O[5_554]	2.17	0.03

5.3 Torsion angles (i)

5.3.1 Protein backbone (i)

In the following table, the Percentiles column shows the percent Ramachandran outliers of the chain as a percentile score with respect to all X-ray entries followed by that with respect to entries of similar resolution.

The Analysed column shows the number of residues for which the backbone conformation was analysed, and the total number of residues.

Mol	Chain	Analysed	Favoured	Allowed	Outliers	Percentiles	
1	A	149/174 (86%)	145 (97%)	4 (3%)	0	100	100
1	B	153/174 (88%)	146 (95%)	7 (5%)	0	100	100

Continued on next page...

CONFIDENTIAL VALIDATION REPORT

Continued from previous page...

Mol	Chain	Analysed	Favoured	Allowed	Outliers	Percentiles	
1	C	149/174 (86%)	146 (98%)	3 (2%)	0	100	100
All	All	451/522 (86%)	437 (97%)	14 (3%)	0	100	100

There are no Ramachandran outliers to report.

5.3.2 Protein sidechains (i)

In the following table, the Percentiles column shows the percent sidechain outliers of the chain as a percentile score with respect to all X-ray entries followed by that with respect to entries of similar resolution.

The Analysed column shows the number of residues for which the sidechain conformation was analysed, and the total number of residues.

Mol	Chain	Analysed	Rotameric	Outliers	Percentiles	
1	A	136/157 (87%)	135 (99%)	1 (1%)	85	74
1	B	140/157 (89%)	139 (99%)	1 (1%)	85	74
1	C	136/157 (87%)	135 (99%)	1 (1%)	85	74
All	All	412/471 (88%)	409 (99%)	3 (1%)	85	74

All (3) residues with a non-rotameric sidechain are listed below:

Mol	Chain	Res	Type
1	B	112	LEU
1	A	89	ARG
1	C	56	TRP

Some sidechains can be flipped to improve hydrogen bonding and reduce clashes. All (2) such sidechains are listed below:

Mol	Chain	Res	Type
1	B	82	GLN
1	C	18	GLN

5.3.3 RNA (i)

There are no RNA molecules in this entry.

CONFIDENTIAL VALIDATION REPORT

5.4 Non-standard residues in protein, DNA, RNA chains (i)

There are no non-standard protein/DNA/RNA residues in this entry.

5.5 Carbohydrates (i)

There are no carbohydrates in this entry.

5.6 Ligand geometry (i)

There are no ligands in this entry.

5.7 Other polymers (i)

There are no such residues in this entry.

5.8 Polymer linkage issues (i)

There are no chain breaks in this entry.

CONFIDENTIAL VALIDATION REPORT

6 Fit of model and data (i)

6.1 Protein, DNA and RNA chains (i)

In the following table, the column labelled '#RSRZ> 2' contains the number (and percentage) of RSRZ outliers, followed by percent RSRZ outliers for the chain as percentile scores relative to all X-ray entries and entries of similar resolution. The OWAB column contains the minimum, median, 95th percentile and maximum values of the occupancy-weighted average B-factor per residue. The column labelled 'Q< 0.9' lists the number of (and percentage) of residues with an average occupancy less than 0.9.

Mol	Chain	Analysed	<RSRZ>	#RSRZ>2			OWAB(Å^2)	Q<0.9
1	A	151/174 (86%)	-0.37	1 (0%)	87	89	16, 21, 39, 67	0
1	B	155/174 (89%)	-0.28	3 (1%)	66	70	17, 23, 38, 49	0
1	C	151/174 (86%)	-0.05	6 (3%)	38	38	17, 25, 42, 66	0
All	All	457/522 (87%)	-0.24	10 (2%)	62	63	16, 23, 40, 67	0

All (10) RSRZ outliers are listed below:

Mol	Chain	Res	Type	RSRZ
1	C	6	ASN	8.3
1	A	6	ASN	7.8
1	C	7	ILE	4.1
1	C	13	PHE	3.5
1	C	8	VAL	3.5
1	B	93	ARG	3.0
1	B	3	LEU	2.7
1	B	2	ASN	2.1
1	C	82	GLN	2.0
1	C	10	GLU	2.0

6.2 Non-standard residues in protein, DNA, RNA chains (i)

There are no non-standard protein/DNA/RNA residues in this entry.

6.3 Carbohydrates (i)

There are no carbohydrates in this entry.

CONFIDENTIAL VALIDATION REPORT

6.4 Ligands (i)

There are no ligands in this entry.

6.5 Other polymers (i)

There are no such residues in this entry.

CONFIDENTIAL VALIDATION REPORT
