# Supplementary material for: Probing the impact of nairovirus genomic diversity on viral ovarian tumor domain protease (vOTU) structure and deubiquitinase activity
Source: PLoS Pathog. 2019 Jan 10;15(1):e1007515. doi: 10.1371/journal.ppat.1007515 (PMC6343935; doi:10.1371/journal.ppat.1007515)
Supplement: S4 File — (PDF) [file ppat.1007515.s008.pdf]

Full wwPDB X-ray Structure Validation Report (i)

CONFIDENTIAL VALIDATION REPORT

Jul 2, 2018 – 11:17 AM EDT

PDB ID	:	6DX3
Title	:	Crystal structure of the viral OTU domain protease from Taggert virus
Deposited on	:	2018-06-28
Resolution	:	2.05 Å(reported)

This is a Full wwPDB X-ray Structure Validation Report.

This report is produced by the wwPDB biocuration pipeline after annotation of the structure.

We welcome your comments at *validation@mail.wwpdb.org*
A user guide is available at
https://www.wwpdb.org/validation/2017/XrayValidationReportHelp
with specific help available everywhere you see the (i) symbol.

The following versions of software and data (see references (i)) were used in the production of this report:

MolProbity	:	4.02b-467
Xtriage (Phenix)	:	1.13
EDS	:	rb-20031172
Percentile statistics	:	20171227.v01 (using entries in the PDB archive December 27th 2017)
Refmac	:	5.8.0158
CCP4	:	7.0 (Gargrove)
Ideal geometry (proteins)	:	Engh & Huber (2001)
Ideal geometry (DNA, RNA)	:	Parkinson et al. (1996)
Validation Pipeline (wwPDB-VP)	:	rb-20031172

1 Overall quality at a glance (i)

The following experimental techniques were used to determine the structure:
X-RAY DIFFRACTION

The reported resolution of this entry is 2.05 Å.

Percentile scores (ranging between 0-100) for global validation metrics of the entry are shown in the following graphic. The table shows the number of entries on which the scores are based.

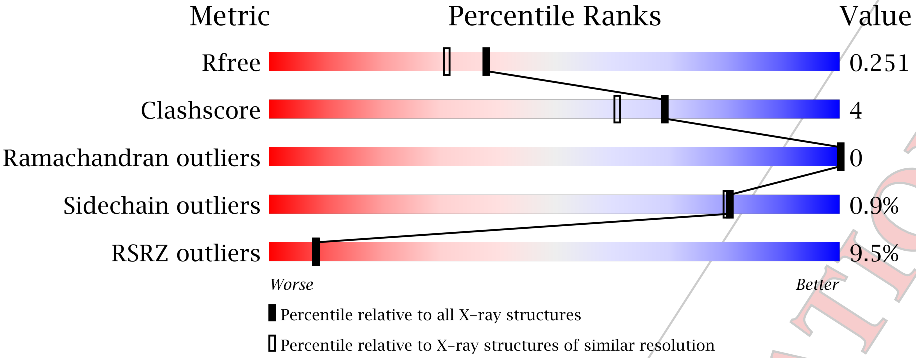

Metric	Whole archive (#Entries)	Similar resolution (#Entries, resolution range(Å))
R_{free}	111664	2250 (2.08-2.04)
Clashscore	122126	2358 (2.08-2.04)
Ramachandran outliers	120053	2339 (2.08-2.04)
Sidechain outliers	120020	2339 (2.08-2.04)
RSRZ outliers	108989	2211 (2.08-2.04)

The table below summarises the geometric issues observed across the polymeric chains and their fit to the electron density. The red, orange, yellow and green segments on the lower bar indicate the fraction of residues that contain outliers for >=3, 2, 1 and 0 types of geometric quality criteria. A grey segment represents the fraction of residues that are not modelled. The numeric value for each fraction is indicated below the corresponding segment, with a dot representing fractions <=5% The upper red bar (where present) indicates the fraction of residues that have poor fit to the electron density. The numeric value is given above the bar.

Mol	Chain	Length	Quality of chain
1	A	175	9% / 87% 5% 8%
1	B	175	10% / 85% 9% 6%
1	C	175	6% / 86% 7% 7%
1	D	175	10% / 87% 6% • 6%

CONFIDENTIAL VALIDATION REPORT

2 Entry composition (i)

There are 3 unique types of molecules in this entry. The entry contains 5775 atoms, of which 0 are hydrogens and 0 are deuteriums.

In the tables below, the ZeroOcc column contains the number of atoms modelled with zero occupancy, the AltConf column contains the number of residues with at least one atom in alternate conformation and the Trace column contains the number of residues modelled with at most 2 atoms.

- Molecule 1 is a protein called RNA-dependent RNA polymerase.

Mol	Chain	Residues	Atoms					ZeroOcc	AltConf	Trace
1	A	161	Total 1329	C 844	N 222	O 255	S 8	0	0	0
1	B	164	Total 1353	C 858	N 226	O 261	S 8	0	0	0
1	C	163	Total 1344	C 853	N 225	O 258	S 8	0	0	0
1	D	164	Total 1353	C 859	N 226	O 259	S 9	0	1	0

There are 36 discrepancies between the modelled and reference sequences:

Chain	Residue	Modelled	Actual	Comment	Reference
A	167	SER	-	expression tag	UNP A0A142J8F6
A	168	GLY	-	expression tag	UNP A0A142J8F6
A	169	SER	-	expression tag	UNP A0A142J8F6
A	170	HIS	-	expression tag	UNP A0A142J8F6
A	171	HIS	-	expression tag	UNP A0A142J8F6
A	172	HIS	-	expression tag	UNP A0A142J8F6
A	173	HIS	-	expression tag	UNP A0A142J8F6
A	174	HIS	-	expression tag	UNP A0A142J8F6
A	175	HIS	-	expression tag	UNP A0A142J8F6
B	167	SER	-	expression tag	UNP A0A142J8F6
B	168	GLY	-	expression tag	UNP A0A142J8F6
B	169	SER	-	expression tag	UNP A0A142J8F6
B	170	HIS	-	expression tag	UNP A0A142J8F6
B	171	HIS	-	expression tag	UNP A0A142J8F6
B	172	HIS	-	expression tag	UNP A0A142J8F6
B	173	HIS	-	expression tag	UNP A0A142J8F6
B	174	HIS	-	expression tag	UNP A0A142J8F6
B	175	HIS	-	expression tag	UNP A0A142J8F6
C	167	SER	-	expression tag	UNP A0A142J8F6
C	168	GLY	-	expression tag	UNP A0A142J8F6
C	169	SER	-	expression tag	UNP A0A142J8F6

Continued on next page...

CONFIDENTIAL VALIDATION REPORT

Continued from previous page...

Chain	Residue	Modelled	Actual	Comment	Reference
C	170	HIS	-	expression tag	UNP A0A142J8F6
C	171	HIS	-	expression tag	UNP A0A142J8F6
C	172	HIS	-	expression tag	UNP A0A142J8F6
C	173	HIS	-	expression tag	UNP A0A142J8F6
C	174	HIS	-	expression tag	UNP A0A142J8F6
C	175	HIS	-	expression tag	UNP A0A142J8F6
D	167	SER	-	expression tag	UNP A0A142J8F6
D	168	GLY	-	expression tag	UNP A0A142J8F6
D	169	SER	-	expression tag	UNP A0A142J8F6
D	170	HIS	-	expression tag	UNP A0A142J8F6
D	171	HIS	-	expression tag	UNP A0A142J8F6
D	172	HIS	-	expression tag	UNP A0A142J8F6
D	173	HIS	-	expression tag	UNP A0A142J8F6
D	174	HIS	-	expression tag	UNP A0A142J8F6
D	175	HIS	-	expression tag	UNP A0A142J8F6

- Molecule 2 is MAGNESIUM ION (three-letter code: MG) (formula: Mg).

Mol	Chain	Residues	Atoms	ZeroOcc	AltConf
2	A	1	Total Mg<br>1 1	0	0
2	D	1	Total Mg<br>1 1	0	0

- Molecule 3 is water.

Mol	Chain	Residues	Atoms	ZeroOcc	AltConf
3	A	110	Total O<br>110 110	0	0
3	B	101	Total O<br>101 101	0	0
3	C	94	Total O<br>94 94	0	0
3	D	89	Total O<br>89 89	0	0

CONFIDENTIAL VALIDATION REPORT

3 Residue-property plots ⓘ

These plots are drawn for all protein, RNA and DNA chains in the entry. The first graphic for a chain summarises the proportions of the various outlier classes displayed in the second graphic. The second graphic shows the sequence view annotated by issues in geometryand electron density. Residues are color-coded according to the number of geometric quality criteria for which they contain at least one outlier: green = 0, yellow = 1, orange = 2 and red = 3 or more. A red dot above a residue indicates a poor fit to the electron density (RSRZ > 2). Stretches of 2 or more consecutive residues without any outlier are shown as a green connector. Residues present in the sample, but not in the model, are shown in grey.

- Molecule 1: RNA-dependent RNA polymerase

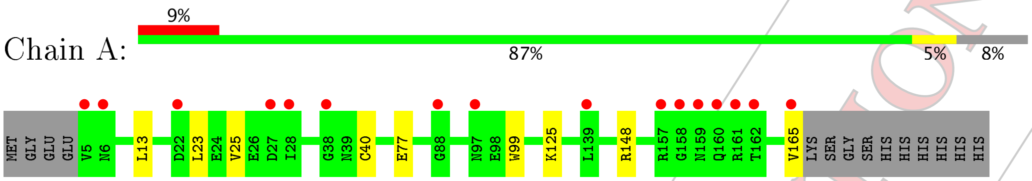

- Molecule 1: RNA-dependent RNA polymerase

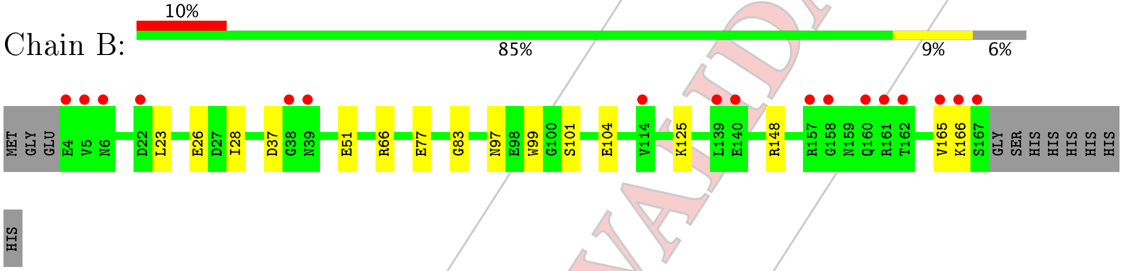

- Molecule 1: RNA-dependent RNA polymerase

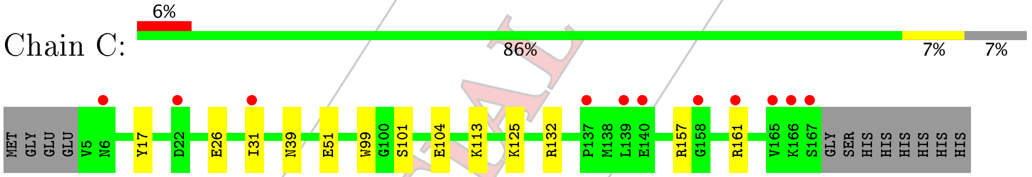

- Molecule 1: RNA-dependent RNA polymerase

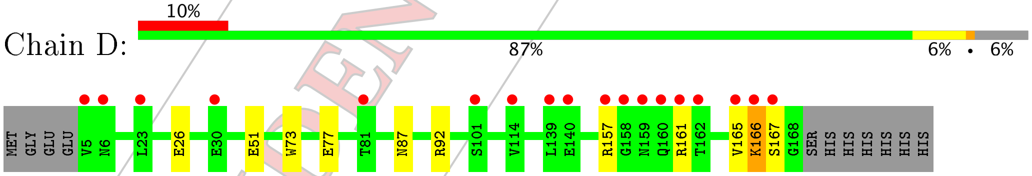

CONFIDENTIAL VALIDATION REPORT

4 Data and refinement statistics

Property	Value	Source
Space group	P 1	Depositor
Cell constants<br>a, b, c, α, β, γ	47.16Å 60.95Å 66.50Å<br>64.95° 89.84° 84.31°	Depositor
Resolution (Å)	36.46 – 2.05<br>36.46 – 2.05	Depositor<br>EDS
% Data completeness<br>(in resolution range)	96.4 (36.46-2.05)<br>96.5 (36.46-2.05)	Depositor<br>EDS
R_{merge}	(Not available)	Depositor
R_{sym}	(Not available)	Depositor
$< I/\sigma(I) >$ [1]	3.77 (at 2.05Å)	Xtriage
Refinement program	PHENIX (1.11.1_2575: ???)	Depositor
R, R_{free}	0.227 , 0.250<br>0.228 , 0.251	Depositor<br>DCC
R_{free} test set	1936 reflections (4.81%)	wwPDB-VP
Wilson B-factor (Å^2)	27.4	Xtriage
Anisotropy	0.562	Xtriage
Bulk solvent k_{sol}(e/Å^3), B_{sol}(Å^2)	0.37 , 52.1	EDS
L-test for twinning[2]	$< \|L\| >$ = 0.49, $< L^2 >$ = 0.33	Xtriage
Estimated twinning fraction	No twinning to report.	Xtriage
F_o,F_c correlation	0.93	EDS
Total number of atoms	5775	wwPDB-VP
Average B, all atoms (Å^2)	35.0	wwPDB-VP

Xtriage's analysis on translational NCS is as follows: *The largest off-origin peak in the Patterson function is 16.35% of the height of the origin peak. No significant pseudotranslation is detected.*

[1] Intensities estimated from amplitudes.

[2] Theoretical values of $< |L| >$, $< L^2 >$ for acentric reflections are 0.5, 0.333 respectively for untwinned datasets, and 0.375, 0.2 for perfectly twinned datasets.

CONFIDENTIAL VALIDATION REPORT

5 Model quality (i)

5.1 Standard geometry (i)

Bond lengths and bond angles in the following residue types are not validated in this section: MG

The Z score for a bond length (or angle) is the number of standard deviations the observed value is removed from the expected value. A bond length (or angle) with $|Z| > 5$ is considered an outlier worth inspection. RMSZ is the root-mean-square of all Z scores of the bond lengths (or angles).

Mol	Chain	Bond lengths		Bond angles	
		RMSZ	$\#\|Z\| > 5$	RMSZ	$\#\|Z\| > 5$
1	A	0.29	0/1364	0.47	0/1851
1	B	0.34	0/1388	0.50	0/1882
1	C	0.31	0/1379	0.49	0/1870
1	D	0.33	0/1391	0.50	0/1885
All	All	0.32	0/5522	0.49	0/7488

There are no bond length outliers.

There are no bond angle outliers.

There are no chirality outliers.

There are no planarity outliers.

5.2 Too-close contacts (i)

In the following table, the Non-H and H(model) columns list the number of non-hydrogen atoms and hydrogen atoms in the chain respectively. The H(added) column lists the number of hydrogen atoms added and optimized by MolProbity. The Clashes column lists the number of clashes within the asymmetric unit, whereas Symm-Clashes lists symmetry related clashes.

Mol	Chain	Non-H	H(model)	H(added)	Clashes	Symm-Clashes
1	A	1329	0	1254	8	0
1	B	1353	0	1278	17	0
1	C	1344	0	1272	9	0
1	D	1353	0	1284	9	0
2	A	1	0	0	0	0
2	D	1	0	0	0	0
3	A	110	0	0	4	1
3	B	101	0	0	11	0
3	C	94	0	0	5	1

Continued on next page...

CONFIDENTIAL VALIDATION REPORT

Continued from previous page...

Mol	Chain	Non-H	H(model)	H(added)	Clashes	Symm-Clashes
3	D	89	0	0	5	0
All	All	5775	0	5088	42	1

The all-atom clashscore is defined as the number of clashes found per 1000 atoms (including hydrogen atoms). The all-atom clashscore for this structure is 4.

All (42) close contacts within the same asymmetric unit are listed below, sorted by their clash magnitude.

Atom-1	Atom-2	Interatomic distance (Å)	Clash overlap (Å)
1:A:13:LEU:HD11	3:C:210:HOH:O	1.47	1.13
1:B:148:ARG:NH1	3:B:201:HOH:O	1.89	1.05
1:C:113:LYS:NZ	3:C:201:HOH:O	1.90	1.02
1:B:99:TRP:NE1	3:B:202:HOH:O	1.97	0.96
1:D:51:GLU:OE2	3:D:301:HOH:O	1.85	0.94
1:A:77:GLU:OE2	3:A:301:HOH:O	1.96	0.81
1:B:99:TRP:CE2	3:B:202:HOH:O	2.37	0.77
1:B:37:ASP:OD2	3:B:203:HOH:O	2.04	0.74
1:B:148:ARG:NH2	3:B:206:HOH:O	2.20	0.74
1:B:51:GLU:OE1	3:B:204:HOH:O	2.13	0.67
1:D:77:GLU:OE2	3:D:303:HOH:O	2.13	0.66
1:B:66:ARG:NH2	3:B:208:HOH:O	2.28	0.65
1:A:99:TRP:HE3	3:A:362:HOH:O	1.80	0.65
1:B:26:GLU:CD	1:B:125:LYS:HE3	2.18	0.64
1:B:83:GLY:O	1:D:166:LYS:HB2	2.01	0.60
1:C:39:ASN:ND2	3:C:203:HOH:O	2.23	0.60
1:D:87:ASN:N	3:D:309:HOH:O	2.33	0.60
1:D:92:ARG:NH1	3:D:307:HOH:O	2.30	0.59
1:A:148:ARG:CZ	3:A:323:HOH:O	2.52	0.58
1:C:157:ARG:NH1	3:C:209:HOH:O	2.36	0.58
1:B:99:TRP:HE3	3:B:270:HOH:O	1.90	0.54
1:D:51:GLU:O	1:D:161:ARG:NH2	2.42	0.52
1:C:31:ILE:HG12	3:C:221:HOH:O	2.09	0.52
1:B:99:TRP:CZ2	3:B:202:HOH:O	2.60	0.50
1:B:77:GLU:OE1	3:B:205:HOH:O	2.20	0.50
1:B:26:GLU:OE1	1:B:125:LYS:HE3	2.14	0.48
1:D:165:VAL:O	1:D:165:VAL:HG23	2.14	0.47
1:B:37:ASP:HB2	1:B:97:ASN:OD1	2.14	0.47
1:D:26:GLU:HB3	1:D:157:ARG:HH21	1.81	0.46
1:D:73:TRP:NE1	3:D:302:HOH:O	2.07	0.46
1:B:23:LEU:HD11	1:B:28:ILE:HD12	1.97	0.46
1:A:40:CYS:SG	1:A:99:TRP:HZ3	2.38	0.45

Continued on next page...

CONFIDENTIAL VALIDATION REPORT

Continued from previous page...

Atom-1	Atom-2	Interatomic distance (Å)	Clash overlap (Å)
1:B:66:ARG:HD2	3:B:229:HOH:O	2.18	0.44
1:B:101:SER:N	1:B:104:GLU:OE2	2.51	0.44
1:A:23:LEU:HD21	1:A:25:VAL:HA	1.99	0.44
1:C:26:GLU:HG3	1:C:125:LYS:HZ1	1.83	0.43
1:A:25:VAL:HG22	1:A:125:LYS:O	2.17	0.43
1:C:26:GLU:HG3	1:C:125:LYS:NZ	2.34	0.43
1:A:165:VAL:O	3:A:302:HOH:O	2.22	0.42
1:C:17:TYR:HB2	1:C:132:ARG:HB3	2.02	0.41
1:C:51:GLU:CD	1:C:161:ARG:HE	2.25	0.40
1:C:101:SER:N	1:C:104:GLU:OE1	2.51	0.40

All (1) symmetry-related close contacts are listed below. The label for Atom-2 includes the symmetry operator and encoded unit-cell translations to be applied.

Atom-1	Atom-2	Interatomic distance (Å)	Clash overlap (Å)
3:A:397:HOH:O	3:C:279:HOH:O[1_655]	2.08	0.12

5.3 Torsion angles (i)

5.3.1 Protein backbone (i)

In the following table, the Percentiles column shows the percent Ramachandran outliers of the chain as a percentile score with respect to all X-ray entries followed by that with respect to entries of similar resolution.

The Analysed column shows the number of residues for which the backbone conformation was analysed, and the total number of residues.

Mol	Chain	Analysed	Favoured	Allowed	Outliers	Percentiles	
1	A	159/175 (91%)	155 (98%)	4 (2%)	0	100	100
1	B	162/175 (93%)	157 (97%)	5 (3%)	0	100	100
1	C	161/175 (92%)	157 (98%)	4 (2%)	0	100	100
1	D	163/175 (93%)	159 (98%)	4 (2%)	0	100	100
All	All	645/700 (92%)	628 (97%)	17 (3%)	0	100	100

There are no Ramachandran outliers to report.

CONFIDENTIAL VALIDATION REPORT

5.3.2 Protein sidechains (i)

In the following table, the Percentiles column shows the percent sidechain outliers of the chain as a percentile score with respect to all X-ray entries followed by that with respect to entries of similar resolution.

The Analysed column shows the number of residues for which the sidechain conformation was analysed, and the total number of residues.

Mol	Chain	Analysed	Rotameric	Outliers	Percentiles	
1	A	145/157 (92%)	145 (100%)	0	100	100
1	B	148/157 (94%)	146 (99%)	2 (1%)	69	67
1	C	147/157 (94%)	146 (99%)	1 (1%)	85	85
1	D	148/157 (94%)	146 (99%)	2 (1%)	69	67
All	All	588/628 (94%)	583 (99%)	5 (1%)	81	80

All (5) residues with a non-rotameric sidechain are listed below:

Mol	Chain	Res	Type
1	B	165	VAL
1	B	166	LYS
1	C	99	TRP
1	D	166	LYS
1	D	167	SER

Some sidechains can be flipped to improve hydrogen bonding and reduce clashes. There are no such sidechains identified.

5.3.3 RNA (i)

There are no RNA molecules in this entry.

5.4 Non-standard residues in protein, DNA, RNA chains (i)

There are no non-standard protein/DNA/RNA residues in this entry.

5.5 Carbohydrates (i)

There are no carbohydrates in this entry.

CONFIDENTIAL VALIDATION REPORT

5.6 Ligand geometry (i)

Of 2 ligands modelled in this entry, 2 are monoatomic - leaving 0 for Mogul analysis.

There are no bond length outliers.

There are no bond angle outliers.

There are no chirality outliers.

There are no torsion outliers.

There are no ring outliers.

No monomer is involved in short contacts.

5.7 Other polymers (i)

There are no such residues in this entry.

5.8 Polymer linkage issues (i)

There are no chain breaks in this entry.

CONFIDENTIAL VALIDATION REPORT

6 Fit of model and data (i)

6.1 Protein, DNA and RNA chains (i)

In the following table, the column labelled '#RSRZ> 2' contains the number (and percentage) of RSRZ outliers, followed by percent RSRZ outliers for the chain as percentile scores relative to all X-ray entries and entries of similar resolution. The OWAB column contains the minimum, median, 95th percentile and maximum values of the occupancy-weighted average B-factor per residue. The column labelled 'Q< 0.9' lists the number of (and percentage) of residues with an average occupancy less than 0.9.

Mol	Chain	Analysed	<RSRZ>	#RSRZ>2			OWAB(Å^2)	Q<0.9
1	A	161/175 (92%)	0.86	16 (9%)	7	7	20, 31, 54, 78	0
1	B	164/175 (93%)	0.77	17 (10%)	6	6	18, 31, 63, 92	0
1	C	163/175 (93%)	0.73	11 (6%)	18	18	21, 31, 57, 78	0
1	D	164/175 (93%)	0.84	18 (10%)	5	5	22, 32, 72, 99	0
All	All	652/700 (93%)	0.80	62 (9%)	8	8	18, 31, 65, 99	0

All (62) RSRZ outliers are listed below:

Mol	Chain	Res	Type	RSRZ
1	D	139	LEU	8.1
1	B	167	SER	6.6
1	B	161	ARG	6.2
1	C	139	LEU	5.9
1	B	165	VAL	5.7
1	C	6	ASN	5.6
1	B	139	LEU	5.5
1	A	139	LEU	5.5
1	A	158	GLY	5.3
1	A	6	ASN	4.8
1	C	161	ARG	4.7
1	A	161	ARG	4.5
1	D	6	ASN	4.2
1	B	5	VAL	4.2
1	A	27	ASP	4.1
1	D	160	GLN	4.0
1	D	167	SER	4.0
1	A	157	ARG	4.0
1	D	165	VAL	3.9
1	D	5	VAL	3.9
1	C	158	GLY	3.9

Continued on next page...

CONFIDENTIAL VALIDATION REPORT

Continued from previous page...

Mol	Chain	Res	Type	RSRZ
1	C	165	VAL	3.8
1	B	6	ASN	3.8
1	A	38	GLY	3.7
1	D	157	ARG	3.6
1	B	140	GLU	3.5
1	A	5	VAL	3.5
1	D	159	ASN	3.5
1	D	162	THR	3.4
1	B	114	VAL	3.4
1	A	160	GLN	3.3
1	A	159	ASN	3.3
1	A	22	ASP	3.3
1	B	4	GLU	3.3
1	D	140	GLU	3.2
1	B	158	GLY	2.8
1	B	166	LYS	2.7
1	D	161	ARG	2.7
1	A	162	THR	2.7
1	D	166	LYS	2.6
1	A	28	ILE	2.6
1	B	162	THR	2.5
1	C	31	ILE	2.5
1	C	22	ASP	2.5
1	D	101	SER	2.4
1	D	158	GLY	2.4
1	B	160	GLN	2.4
1	A	97	ASN	2.3
1	D	23	LEU	2.3
1	C	167	SER	2.3
1	B	39	ASN	2.3
1	B	157	ARG	2.3
1	D	81	THR	2.2
1	D	114	VAL	2.2
1	A	88	GLY	2.1
1	B	38	GLY	2.1
1	D	30	GLU	2.1
1	C	137	PRO	2.1
1	B	22	ASP	2.1
1	A	165	VAL	2.0
1	C	140	GLU	2.0
1	C	166	LYS	2.0

CONFIDENTIAL VALIDATION REPORT

6.2 Non-standard residues in protein, DNA, RNA chains (i)

There are no non-standard protein/DNA/RNA residues in this entry.

6.3 Carbohydrates (i)

There are no carbohydrates in this entry.

6.4 Ligands (i)

In the following table, the Atoms column lists the number of modelled atoms in the group and the number defined in the chemical component dictionary. The B-factors column lists the minimum, median, 95th percentile and maximum values of B factors of atoms in the group. The column labelled 'Q< 0.9' lists the number of atoms with occupancy less than 0.9.

Mol	Type	Chain	Res	Atoms	RSCC	RSR	B-factors($Å^2$)	Q<0.9
2	MG	D	201	1/1	0.76	0.30	54,54,54,54	0
2	MG	A	201	1/1	0.92	0.07	56,56,56,56	0

6.5 Other polymers (i)

There are no such residues in this entry.

CONFIDENTIAL VALIDATION REPORT
