# Supplementary material for: Probing the impact of nairovirus genomic diversity on viral ovarian tumor domain protease (vOTU) structure and deubiquitinase activity
Source: PLoS Pathog. 2019 Jan 10;15(1):e1007515. doi: 10.1371/journal.ppat.1007515 (PMC6343935; doi:10.1371/journal.ppat.1007515)
Supplement: S5 File — (PDF) [file ppat.1007515.s009.pdf]

Full wwPDB X-ray Structure Validation Report (i)

Jul 2, 2018 – 11:09 AM EDT

PDB ID	:	6DX5
Title	:	Crystal structure of the viral OTU domain protease from Farallon virus
Deposited on	:	2018-06-28
Resolution	:	2.22 Å(reported)

This is a Full wwPDB X-ray Structure Validation Report.

This report is produced by the wwPDB biocuration pipeline after annotation of the structure.

We welcome your comments at *validation@mail.wwpdb.org*
A user guide is available at
https://www.wwpdb.org/validation/2017/XrayValidationReportHelp
with specific help available everywhere you see the (i) symbol.

CONFIDENTIAL VALIDATION REPORT

The following versions of software and data (see references (i)) were used in the production of this report:

MolProbity	:	4.02b-467
Mogul	:	1.7.3 (157068), CSD as539be (2018)
Xtriage (Phenix)	:	1.13
EDS	:	rb-20031172
Percentile statistics	:	20171227.v01 (using entries in the PDB archive December 27th 2017)
Refmac	:	5.8.0158
CCP4	:	7.0 (Gargrove)
Ideal geometry (proteins)	:	Engh & Huber (2001)
Ideal geometry (DNA, RNA)	:	Parkinson et al. (1996)
Validation Pipeline (wwPDB-VP)	:	rb-20031172

1 Overall quality at a glance (i)

The following experimental techniques were used to determine the structure:
X-RAY DIFFRACTION

The reported resolution of this entry is 2.22 Å.

Percentile scores (ranging between 0-100) for global validation metrics of the entry are shown in the following graphic. The table shows the number of entries on which the scores are based.

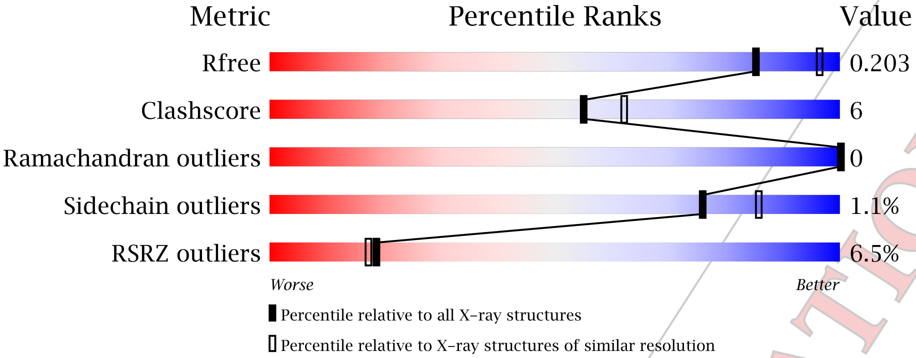

Metric	Whole archive (#Entries)	Similar resolution (#Entries, resolution range(Å))
R_{free}	111664	5192 (2.24-2.20)
Clashscore	122126	5911 (2.24-2.20)
Ramachandran outliers	120053	5828 (2.24-2.20)
Sidechain outliers	120020	5829 (2.24-2.20)
RSRZ outliers	108989	5078 (2.24-2.20)

The table below summarises the geometric issues observed across the polymeric chains and their fit to the electron density. The red, orange, yellow and green segments on the lower bar indicate the fraction of residues that contain outliers for >=3, 2, 1 and 0 types of geometric quality criteria. A grey segment represents the fraction of residues that are not modelled. The numeric value for each fraction is indicated below the corresponding segment, with a dot representing fractions <=5% The upper red bar (where present) indicates the fraction of residues that have poor fit to the electron density. The numeric value is given above the bar.

Mol	Chain	Length	Quality of chain
1	A	203	6% / 72% 11% 17%
1	B	203	4% / 73% 10% 17%

CONFIDENTIAL VALIDATION REPORT

2 Entry composition (i)

There are 3 unique types of molecules in this entry. The entry contains 2923 atoms, of which 0 are hydrogens and 0 are deuteriums.

In the tables below, the ZeroOcc column contains the number of atoms modelled with zero occupancy, the AltConf column contains the number of residues with at least one atom in alternate conformation and the Trace column contains the number of residues modelled with at most 2 atoms.

- Molecule 1 is a protein called RNA-dependent RNA polymerase.

Mol	Chain	Residues	Atoms					ZeroOcc	AltConf	Trace
			Total	C	N	O	S			
1	B	169	1365	872	231	255	7	0	0	0
1	A	169	1365	872	231	255	7	0	0	0

There are 18 discrepancies between the modelled and reference sequences:

Chain	Residue	Modelled	Actual	Comment	Reference
B	195	SER	-	expression tag	UNP A0A191KW96
B	196	GLY	-	expression tag	UNP A0A191KW96
B	197	SER	-	expression tag	UNP A0A191KW96
B	198	HIS	-	expression tag	UNP A0A191KW96
B	199	HIS	-	expression tag	UNP A0A191KW96
B	200	HIS	-	expression tag	UNP A0A191KW96
B	201	HIS	-	expression tag	UNP A0A191KW96
B	202	HIS	-	expression tag	UNP A0A191KW96
B	203	HIS	-	expression tag	UNP A0A191KW96
A	195	SER	-	expression tag	UNP A0A191KW96
A	196	GLY	-	expression tag	UNP A0A191KW96
A	197	SER	-	expression tag	UNP A0A191KW96
A	198	HIS	-	expression tag	UNP A0A191KW96
A	199	HIS	-	expression tag	UNP A0A191KW96
A	200	HIS	-	expression tag	UNP A0A191KW96
A	201	HIS	-	expression tag	UNP A0A191KW96
A	202	HIS	-	expression tag	UNP A0A191KW96
A	203	HIS	-	expression tag	UNP A0A191KW96

- Molecule 2 is 2,3-DIHYDROXY-1,4-DITHIOBUTANE (three-letter code: DTT) (formula: $C_4H_{10}O_2S_2$).

CONFIDENTIAL VALIDATION REPORT

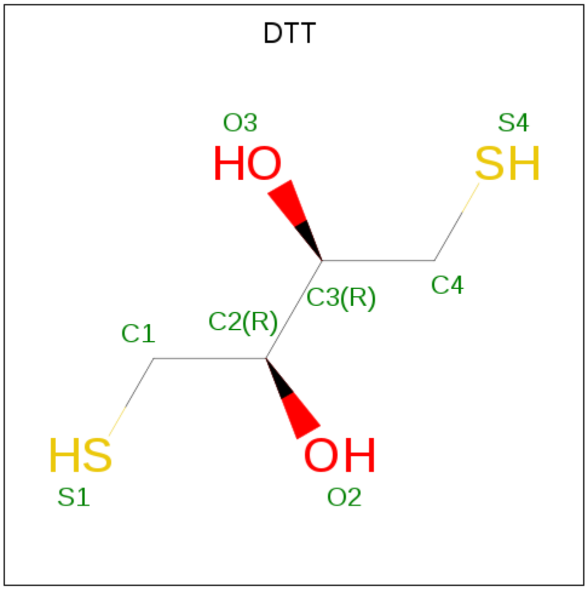

Mol	Chain	Residues	Atoms	ZeroOcc	AltConf
2	B	1	Total C O S<br>8 4 2 2	0	0
2	B	1	Total C O S<br>8 4 2 2	0	0
2	A	1	Total C O S<br>8 4 2 2	0	0

- Molecule 3 is water.

Mol	Chain	Residues	Atoms	ZeroOcc	AltConf
3	B	87	Total O<br>87 87	0	0
3	A	82	Total O<br>82 82	0	0

CONFIDENTIAL VALIDATION REPORT

3 Residue-property plots (i)

These plots are drawn for all protein, RNA and DNA chains in the entry. The first graphic for a chain summarises the proportions of the various outlier classes displayed in the second graphic. The second graphic shows the sequence view annotated by issues in geometryand electron density. Residues are color-coded according to the number of geometric quality criteria for which they contain at least one outlier: green = 0, yellow = 1, orange = 2 and red = 3 or more. A red dot above a residue indicates a poor fit to the electron density (RSRZ > 2). Stretches of 2 or more consecutive residues without any outlier are shown as a green connector. Residues present in the sample, but not in the model, are shown in grey.

- Molecule 1: RNA-dependent RNA polymerase

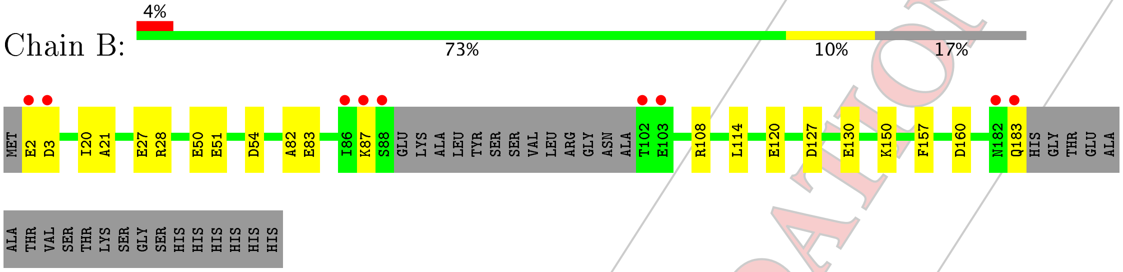

- Molecule 1: RNA-dependent RNA polymerase

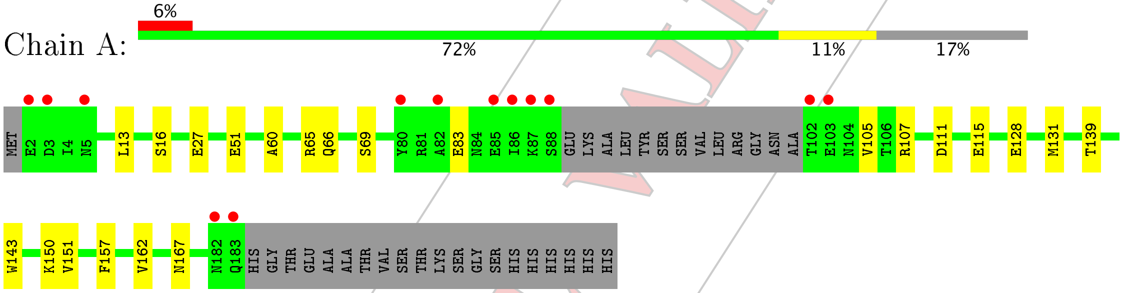

CONFIDENTIAL VALIDATION REPORT

4 Data and refinement statistics (i)

Property	Value	Source
Space group	P 41 21 2	Depositor
Cell constants<br>a, b, c, α, β, γ	70.28Å 70.28Å 176.26Å<br>90.00° 90.00° 90.00°	Depositor
Resolution (Å)	37.94 – 2.22<br>37.94 – 2.22	Depositor<br>EDS
% Data completeness<br>(in resolution range)	99.6 (37.94-2.22)<br>99.6 (37.94-2.22)	Depositor<br>EDS
R_{merge}	(Not available)	Depositor
R_{sym}	(Not available)	Depositor
$< I/\sigma(I) >$ [1]	3.31 (at 2.22Å)	Xtriage
Refinement program	PHENIX (1.10.1_2155: ???)	Depositor
R, R_{free}	0.163 , 0.203<br>0.163 , 0.203	Depositor<br>DCC
R_{free} test set	1136 reflections (5.06%)	wwPDB-VP
Wilson B-factor (Å^2)	31.0	Xtriage
Anisotropy	0.570	Xtriage
Bulk solvent k_{sol}(e/Å^3), B_{sol}(Å^2)	0.34 , 46.6	EDS
L-test for twinning[2]	$< \|L\| > = 0.48$, $< L^2 > = 0.32$	Xtriage
Estimated twinning fraction	No twinning to report.	Xtriage
F_o,F_c correlation	0.96	EDS
Total number of atoms	2923	wwPDB-VP
Average B, all atoms (Å^2)	44.0	wwPDB-VP

Xtriage's analysis on translational NCS is as follows: *The analyses of the Patterson function reveals a significant off-origin peak that is 21.37 % of the origin peak, indicating pseudo-translational symmetry. The chance of finding a peak of this or larger height randomly in a structure without pseudo-translational symmetry is equal to 7.1118e-03. The detected translational NCS is most likely also responsible for the elevated intensity ratio.*

[1] Intensities estimated from amplitudes.

[2] Theoretical values of $< |L| >$, $< L^2 >$ for acentric reflections are 0.5, 0.333 respectively for untwinned datasets, and 0.375, 0.2 for perfectly twinned datasets.

CONFIDENTIAL VALIDATION REPORT

5 Model quality (i)

5.1 Standard geometry (i)

Bond lengths and bond angles in the following residue types are not validated in this section: DTT

The Z score for a bond length (or angle) is the number of standard deviations the observed value is removed from the expected value. A bond length (or angle) with $|Z| > 5$ is considered an outlier worth inspection. RMSZ is the root-mean-square of all Z scores of the bond lengths (or angles).

Mol	Chain	Bond lengths		Bond angles	
		RMSZ	$\#\|Z\| > 5$	RMSZ	$\#\|Z\| > 5$
1	A	0.69	0/1397	0.70	0/1890
1	B	0.76	3/1397 (0.2%)	0.77	1/1890 (0.1%)
All	All	0.72	3/2794 (0.1%)	0.74	1/3780 (0.0%)

All (3) bond length outliers are listed below:

Mol	Chain	Res	Type	Atoms	Z	Observed(Å)	Ideal(Å)
1	B	51	GLU	CG-CD	6.78	1.62	1.51
1	B	51	GLU	CB-CG	5.41	1.62	1.52
1	B	50	GLU	CG-CD	5.14	1.59	1.51

All (1) bond angle outliers are listed below:

Mol	Chain	Res	Type	Atoms	Z	Observed(^{o})	Ideal(^{o})
1	B	28	ARG	NE-CZ-NH1	-5.46	117.57	120.30

There are no chirality outliers.

There are no planarity outliers.

5.2 Too-close contacts (i)

In the following table, the Non-H and H(model) columns list the number of non-hydrogen atoms and hydrogen atoms in the chain respectively. The H(added) column lists the number of hydrogen atoms added and optimized by MolProbity. The Clashes column lists the number of clashes within the asymmetric unit, whereas Symm-Clashes lists symmetry related clashes.

Mol	Chain	Non-H	H(model)	H(added)	Clashes	Symm-Clashes
1	A	1365	0	1305	17	0
1	B	1365	0	1305	12	0

Continued on next page...

CONFIDENTIAL VALIDATION REPORT

Continued from previous page...

Mol	Chain	Non-H	H(model)	H(added)	Clashes	Symm-Clashes
2	A	8	0	10	0	0
2	B	16	0	20	1	0
3	A	82	0	0	4	0
3	B	87	0	0	5	0
All	All	2923	0	2640	30	0

The all-atom clashscore is defined as the number of clashes found per 1000 atoms (including hydrogen atoms). The all-atom clashscore for this structure is 6.

All (30) close contacts within the same asymmetric unit are listed below, sorted by their clash magnitude.

Atom-1	Atom-2	Interatomic distance (Å)	Clash overlap (Å)
1:A:128:GLU:HG3	1:A:131:MET:HE1	1.49	0.94
1:A:143:TRP:HE1	1:A:167:ASN:HD22	1.18	0.91
1:B:2:GLU:OE2	1:B:3:ASP:OD1	1.97	0.83
1:A:60:ALA:HB1	1:A:131:MET:HE2	1.61	0.82
2:B:302:DTT:S4	3:B:439:HOH:O	2.37	0.80
1:A:27:GLU:OE2	3:A:401:HOH:O	2.01	0.77
1:A:66:GLN:NE2	3:A:402:HOH:O	2.11	0.77
1:B:120:GLU:OE1	3:B:401:HOH:O	2.02	0.76
1:A:111:ASP:O	1:A:115:GLU:HG3	1.88	0.73
1:B:160:ASP:OD2	3:B:402:HOH:O	2.10	0.70
1:B:127:ASP:OD1	3:B:403:HOH:O	2.15	0.64
1:A:107:ARG:NH2	1:A:111:ASP:OD2	2.31	0.64
1:A:60:ALA:HB1	1:A:131:MET:CE	2.30	0.59
1:A:65:ARG:NH2	3:A:405:HOH:O	2.38	0.56
1:A:150:LYS:HE2	1:A:151:VAL:O	2.06	0.56
1:A:83:GLU:HG3	1:A:105:VAL:HG21	1.88	0.55
1:A:13:LEU:HD13	1:A:157:PHE:CE1	2.45	0.52
1:B:150:LYS:HE2	1:B:150:LYS:N	2.26	0.51
1:B:83:GLU:O	1:B:87:LYS:HG2	2.10	0.51
1:A:143:TRP:HE1	1:A:167:ASN:ND2	1.98	0.51
1:B:82:ALA:HB1	1:B:108:ARG:HB3	1.94	0.50
1:A:107:ARG:CZ	1:A:111:ASP:OD2	2.60	0.49
1:B:54:ASP:OD2	3:B:404:HOH:O	2.19	0.49
1:B:130:GLU:HB2	1:B:157:PHE:CE1	2.50	0.47
1:A:162:VAL:HG13	1:A:167:ASN:HD21	1.82	0.45
1:A:16:SER:O	3:A:403:HOH:O	2.21	0.44
1:B:20:ILE:HG22	1:B:21:ALA:N	2.33	0.43
1:A:139:THR:HA	1:A:157:PHE:O	2.21	0.41
1:B:114:LEU:HA	1:B:114:LEU:HD23	1.90	0.40

Continued on next page...

CONFIDENTIAL VALIDATION REPORT

Continued from previous page...

Atom-1	Atom-2	Interatomic distance (Å)	Clash overlap (Å)
1:B:183:GLN:OE1	1:B:183:GLN:N	2.55	0.40

There are no symmetry-related clashes.

5.3 Torsion angles (i)

5.3.1 Protein backbone (i)

In the following table, the Percentiles column shows the percent Ramachandran outliers of the chain as a percentile score with respect to all X-ray entries followed by that with respect to entries of similar resolution.

The Analysed column shows the number of residues for which the backbone conformation was analysed, and the total number of residues.

Mol	Chain	Analysed	Favoured	Allowed	Outliers	Percentiles	
1	A	165/203 (81%)	163 (99%)	2 (1%)	0	100	100
1	B	165/203 (81%)	163 (99%)	2 (1%)	0	100	100
All	All	330/406 (81%)	326 (99%)	4 (1%)	0	100	100

There are no Ramachandran outliers to report.

5.3.2 Protein sidechains (i)

In the following table, the Percentiles column shows the percent sidechain outliers of the chain as a percentile score with respect to all X-ray entries followed by that with respect to entries of similar resolution.

The Analysed column shows the number of residues for which the sidechain conformation was analysed, and the total number of residues.

Mol	Chain	Analysed	Rotameric	Outliers	Percentiles	
1	A	142/169 (84%)	140 (99%)	2 (1%)	69	81
1	B	142/169 (84%)	141 (99%)	1 (1%)	85	92
All	All	284/338 (84%)	281 (99%)	3 (1%)	76	86

All (3) residues with a non-rotameric sidechain are listed below:

CONFIDENTIAL VALIDATION REPORT

Mol	Chain	Res	Type
1	B	27	GLU
1	A	51	GLU
1	A	69	SER

Some sidechains can be flipped to improve hydrogen bonding and reduce clashes. All (8) such sidechains are listed below:

Mol	Chain	Res	Type
1	B	36	ASN
1	B	66	GLN
1	B	67	HIS
1	B	84	ASN
1	A	84	ASN
1	A	104	ASN
1	A	144	ASN
1	A	167	ASN

5.3.3 RNA (i)

There are no RNA molecules in this entry.

5.4 Non-standard residues in protein, DNA, RNA chains (i)

There are no non-standard protein/DNA/RNA residues in this entry.

5.5 Carbohydrates (i)

There are no carbohydrates in this entry.

5.6 Ligand geometry (i)

3 ligands are modelled in this entry.

In the following table, the Counts columns list the number of bonds (or angles) for which Mogul statistics could be retrieved, the number of bonds (or angles) that are observed in the model and the number of bonds (or angles) that are defined in the Chemical Component Dictionary. The Link column lists molecule types, if any, to which the group is linked. The Z score for a bond length (or angle) is the number of standard deviations the observed value is removed from the expected value. A bond length (or angle) with $|Z| > 2$ is considered an outlier worth inspection. RMSZ is the root-mean-square of all Z scores of the bond lengths (or angles).

CONFIDENTIAL VALIDATION REPORT

Mol	Type	Chain	Res	Link	Bond lengths			Bond angles		
					Counts	RMSZ	#|Z| > 2	Counts	RMSZ	#|Z| > 2
2	DTT	A	301	-	7,7,7	0.76	0	4,8,8	2.06	1 (25%)
2	DTT	B	301	-	7,7,7	0.92	0	4,8,8	0.94	0
2	DTT	B	302	-	7,7,7	0.83	0	4,8,8	2.09	1 (25%)

In the following table, the Chirals column lists the number of chiral outliers, the number of chiral centers analysed, the number of these observed in the model and the number defined in the Chemical Component Dictionary. Similar counts are reported in the Torsion and Rings columns. '-' means no outliers of that kind were identified.

Mol	Type	Chain	Res	Link	Chirals	Torsions	Rings
2	DTT	A	301	-	-	0/8/8/8	0/0/0/0
2	DTT	B	301	-	-	0/8/8/8	0/0/0/0
2	DTT	B	302	-	-	0/8/8/8	0/0/0/0

There are no bond length outliers.

All (2) bond angle outliers are listed below:

Mol	Chain	Res	Type	Atoms	Z	Observed(^{o})	Ideal(^{o})
2	A	301	DTT	C2-C1-S1	-3.55	103.71	114.45
2	B	302	DTT	C3-C4-S4	-3.53	103.77	114.45

There are no chirality outliers.

There are no torsion outliers.

There are no ring outliers.

1 monomer is involved in 1 short contact:

Mol	Chain	Res	Type	Clashes	Symm-Clashes
2	B	302	DTT	1	0

5.7 Other polymers (i)

There are no such residues in this entry.

5.8 Polymer linkage issues (i)

There are no chain breaks in this entry.

CONFIDENTIAL VALIDATION REPORT

6 Fit of model and data (i)

6.1 Protein, DNA and RNA chains (i)

In the following table, the column labelled '#RSRZ> 2' contains the number (and percentage) of RSRZ outliers, followed by percent RSRZ outliers for the chain as percentile scores relative to all X-ray entries and entries of similar resolution. The OWAB column contains the minimum, median, 95th percentile and maximum values of the occupancy-weighted average B-factor per residue. The column labelled 'Q< 0.9' lists the number of (and percentage) of residues with an average occupancy less than 0.9.

Mol	Chain	Analysed	<RSRZ>	#RSRZ>2			OWAB(Å^2)	Q<0.9
1	A	169/203 (83%)	0.15	13 (7%)	13	12	19, 39, 95, 114	0
1	B	169/203 (83%)	0.01	9 (5%)	26	24	20, 34, 79, 115	0
All	All	338/406 (83%)	0.08	22 (6%)	19	17	19, 36, 93, 115	0

All (22) RSRZ outliers are listed below:

Mol	Chain	Res	Type	RSRZ
1	A	86	ILE	8.9
1	A	183	GLN	7.4
1	B	182	ASN	5.7
1	A	182	ASN	5.5
1	A	88	SER	5.0
1	A	87	LYS	5.0
1	B	102	THR	4.8
1	B	2	GLU	4.8
1	B	3	ASP	4.8
1	A	103	GLU	4.1
1	B	183	GLN	3.9
1	A	102	THR	3.7
1	A	3	ASP	3.7
1	B	103	GLU	3.4
1	B	86	ILE	3.3
1	B	88	SER	3.2
1	A	2	GLU	3.0
1	A	80	TYR	3.0
1	A	85	GLU	2.6
1	B	87	LYS	2.6
1	A	82	ALA	2.3
1	A	5	ASN	2.1

CONFIDENTIAL VALIDATION REPORT

6.2 Non-standard residues in protein, DNA, RNA chains (i)

There are no non-standard protein/DNA/RNA residues in this entry.

6.3 Carbohydrates (i)

There are no carbohydrates in this entry.

6.4 Ligands (i)

In the following table, the Atoms column lists the number of modelled atoms in the group and the number defined in the chemical component dictionary. The B-factors column lists the minimum, median, 95th percentile and maximum values of B factors of atoms in the group. The column labelled 'Q< 0.9' lists the number of atoms with occupancy less than 0.9.

Mol	Type	Chain	Res	Atoms	RSCC	RSR	B-factors(Å^2)	Q<0.9
2	DTT	B	301	8/8	0.59	0.26	100,102,108,114	0
2	DTT	A	301	8/8	0.68	0.25	88,97,112,115	0
2	DTT	B	302	8/8	0.80	0.26	91,103,107,113	0

6.5 Other polymers (i)

There are no such residues in this entry.

CONFIDENTIAL VALIDATION REPORT
